# Detecting consistent patterns of directional adaptation using differential selection codon models

**DOI:** 10.1186/s12862-017-0979-y

**Published:** 2017-06-23

**Authors:** Sahar Parto, Nicolas Lartillot

**Affiliations:** 1 Département de Biochimie et Médecine Moléculaire, Centre Robert Cedergren, Bio-Informatique et Génomique, Université de Montréal, Montréal, Québec, Canada; 2 Laboratoire de Biométrie et Biologie Évolutive, Université Lyon 1, CNRS, UMR 5558, Lyon, France

**Keywords:** HIV, Evolution, Selection, HLA, Virus adaptation, Bayesian, MCMC

## Abstract

**Background:**

Phylogenetic codon models are often used to characterize the selective regimes acting on protein-coding sequences. Recent methodological developments have led to models explicitly accounting for the interplay between mutation and selection, by modeling the amino acid fitness landscape along the sequence. However, thus far, most of these models have assumed that the fitness landscape is constant over time. Fluctuations of the fitness landscape may often be random or depend on complex and unknown factors. However, some organisms may be subject to systematic changes in selective pressure, resulting in reproducible molecular adaptations across independent lineages subject to similar conditions.

**Results:**

Here, we introduce a codon-based differential selection model, which aims to detect and quantify the fine-grained consistent patterns of adaptation at the protein-coding level, as a function of external conditions experienced by the organism under investigation. The model parameterizes the global mutational pressure, as well as the site- and condition-specific amino acid selective preferences. This phylogenetic model is implemented in a Bayesian MCMC framework. After validation with simulations, we applied our method to a dataset of HIV sequences from patients with known HLA genetic background. Our differential selection model detects and characterizes differentially selected coding positions specifically associated with two different HLA alleles.

**Conclusion:**

Our differential selection model is able to identify consistent molecular adaptations as a function of repeated changes in the environment of the organism. These models can be applied to many other problems, ranging from viral adaptation to evolution of life-history strategies in plants or animals.

**Electronic supplementary material:**

The online version of this article (doi:10.1186/s12862-017-0979-y) contains supplementary material, which is available to authorized users.

## Background

Statistical models of molecular evolutionary processes are now widely used to analyze the interplay between mutation and selection. Often, these models are formulated at the codon level, thus relying on the contrast between synonymous and non-synonymous substitutions to leverage out an estimation of the strength of selection acting at various levels (nucleotide, amino acids, codon usage) of protein-coding sequences.

The first codon models, proposed independently by Goldman and Yang [1] and Muse and Gaut [2], relied on a simple aggregate parameter, ω = *dN/dS*, to capture the overall strength of selection, globally over the protein-coding sequence and over the phylogenetic tree. Subsequent elaborations on these original models allowed for variation in *dN/dS* among sites [3, 4] or among lineages [5], or both [6, 7], thus increasing the sensitivity and the resolution of the detection of selective regimes. However, all of these models still do not discriminate between alternative amino acids. Instead, they essentially put all non-synonymous substitutions on the same level [8].

In this direction, Halpern and Bruno [9] and also Thorne et al. [10] have proposed an alternative codon modelling strategy, allowing for site- and amino acid-specific selective effects. The model of Halpern and Bruno also has a clear mechanistic interpretation, being derived from first principles of population genetics. Specifically, the rate of substitution between codons is seen as the product of the mutation rate and the fixation probability. In turn, the fixation probability is made explicitly dependent on the selection coefficient of the mutation under consideration. Selection coefficients are obtained from an explicit fitness landscape, in which the fitness of each amino acid is allowed to be different at each coding site. Technically, the model therefore invokes, at each coding site, a normalized vector of 20 amino acid fitness coefficients, collectively referred to as the site-specific fitness profile. In the original version of Halpern and Bruno [9], site-specific amino acid fitness profiles were empirically estimated based on observed amino acid frequencies. Since then, a statistically more sophisticated version of this model was developed in a Bayesian framework by Rodrigue et al. [8], using a non-parametric approach to integrate over the uncertainty about site-specific selective features (now seen as random-effects across sites), and to capture the unknown law of amino acid fitness profiles across sites. The importance of accounting for modulation of selection across sites by introducing site-specific amino acid fitness profiles was demonstrated by Bayes factor computation and posterior-predictive tests [8]. Of note, more phenomenological variants of this modeling approach, also with site-specific amino acid fitness contributions but without the population-genetic justification of Halpern and Bruno’s paradigm, have been explored [8–11].

This modeling approach, although fairly complex, still leaves an important aspect of protein evolution aside, by assuming that the fitness landscape is constant through time. Yet, many ecological situations clearly suggest that fitness landscapes undergo important fluctuations through time [12]. Two alternative approaches are possible, to relax this specific assumption. First, fluctuations of the fitness landscape could be modelled as a purely latent effect (e.g., Markov-modulated models) [13], thus without relying on any extra information about the environmental or ecological drivers of the fluctuations. Secondly, in some situations, empirical knowledge is available, in terms of varying conditions across sampled genetic sequences. In this context, it is, in principle, possible to explicitly model condition-specific amino acid fitness modulations. The present work is an attempt at modeling such effects.

A clear-cut example where robust empirical knowledge about varying selective environments is available is the evolution of viral sequences as a function of the genetic background represented by the hosts. For example, the analysis of patterns of selection, using *dN/dS* codon models in a phylogenetic maximum likelihood framework, has shown the substantial role of fluctuating selection in the emergence of new mutations and the ability of HIV-1 to escape from immune system [14, 15]. HIV-1 is capable of evading the CTL (Cytotoxic T-Lymphocyte) response because of its rapid rate of mutation in HLA-restricted epitopes, called escape mutation. Escape mutation gives the virus the ability to adapt under different selective forces in different individuals and in response to drugs [16], which makes the design of a vaccine very difficult.

Therefore, understanding the evolution of HIV-1 within the human body, which is both rapid and under strong selection, helps designing more effective vaccines against HIV-1 and control its evolution. On the other hand, the high rate of mutation of HIV-1 enables the virus to produce genetically diverse population in each host, called quasi-species [17], which makes it possible for the virus to adapt to its host even within a single round of infection. In this direction, the correlation between HLA alleles and HIV polymorphisms has been paid a lot of attention in recent years, from population-based studies [18–20] to studies taking phylogeny into account [21, 22]. A method, called the Phylogeny Dependency Network, was introduced to analyze HLA-mediated escape in HIV-1 [23]. This method accounts for the phylogeny, the correlation between coding sites and linkage disequilibrium between HLA alleles. On the other hand, it only takes the information of the tips of the phylogenetic tree into account. More fundamentally, it does not rely on an explicit model of the underlying molecular evolutionary processes. Another phylogenetic model has been used by Tamuri et al. [24] to identify host dependent selective constraints for viruses. These authors specified different host-dependent substitution rates along the phylogenetic tree, and used a maximum likelihood approach, combined with a likelihood-ratio test, to identify positions under differential selection between hosts. This method, first formulated directly at the amino acid level, was then generalized to account for the coding structure [25, 26].

Here, we introduce a codon model able to capture site- and condition-specific amino acid fitness effects. In this differential selection (DS) model, which is implemented in a Bayesian inference framework, a site and branch heterogeneous selection factor is invoked to estimate the substitution rate at the codon level of aligned HIV-1 sequence. As the population-genetics of viral populations is complex and difficult to model quantitatively, we explored two alternative strategies for deriving the codon substitution process, either using a phenomenological approach, or using a mechanistic derivation as in Halpern and Bruno. Our DS model was then used to investigate how the fluctuating environment provided by the diversity of human HLA background affects HIV-1 sequence evolution. We illustrate how our approach finds consistent patterns of viral adaptation, in terms of how selection acts at specific positions, modulating amino acid preference as a function of the HLA background.

## Methods

### HIV-1 data

A dataset of 333 Gag sequences (443 codons) of HIV-1 subtype B from 41 HIV-infected individuals with known HLA types was obtained from the Los Alamos National Laboratory (LANL) HIV database (www.hiv.lanl.gov). Each patient is represented by 8 sequences on average. Information about the HLA types of the patients was also downloaded. About 35% of the sequences are from HLA B57+ patients (the dataset is available in Additional file 1: Table S1). Recombinant sequences were excluded from the study by choosing an internal option in the LANL HIV databases to remove all known CRFs (Circulating Recombinant Forms). The amino acid alignment of the sequences provided by the source was downloaded, manually corrected (misplaced amino acids were relocated and misaligned regions were deleted) and used for back aligning the DNA sequences at the codon level.

### Phylogenetic tree estimation

Primarily for computational reasons, the method introduced here assumes a fixed tree topology. However, owing to the relatively short length of the coding sequences, the tree topology may not be known with high confidence. In addition, there is the question of whether the sequences corresponding to a given patient should form a monophyletic group. This may not always be the case, in particular because of tree reconstruction errors, a problem which can be alleviated simply by constraining the monophyly of each patient during the tree reconstruction. However, non-monophyly could also be real, being caused by complicated multiple infection patterns between individuals. In this case, constraining the monophyly might result in mis-specification of the reconstructed tree topology.

To check the robustness of our method to these potential sources of error, we tested alternative methods for reconstructing the phylogenetic tree and conducted independent analyses under these alternative tree topologies. Specifically, a first tree topology (T1) was obtained directly from the LANL website. This tree was estimated using the neighbor joining algorithm [27]. A second tree (T2) was reconstructed using MrBayes (version 3.2.6) [28, 29], under the GTR + Γ substitution model and constraining the monophyly of the groups corresponding to sequences belonging to a given patient. A third tree (T3) was estimated, still using MrBayes, under the same substitution model, but without imposing any constraint on the tree topology. In MrBayes, we ran MCMC chain for 1,500,000 cycles (the average standard deviation of split frequencies reaches the value less than 0.05, and the Potential Scale Reduction Factor (PSRF) [30], which should approach 1.0 as the two runs converge, was equal to 1.001 and 1.000 for the two chains).

In the case of tree T1 and T3, we observed 20 and 23 cases of non-monophyletic patients, respectively. In both cases, we applied a greedy algorithm for excluding the smallest possible set of sequences such that each patient is then represented by a monophyletic group of sequences. This was done using the following recursive procedure: first, the number of sequences from each host pending from each node was determined recursively at each node, from the tips toward the root. During this recursive scan, wherever a group pending from a given node was not monophyletic, the sequences belonging to the host with the smallest number of sequences pending from that node were flagged. Finally, in a backward recursive scan of the tree, from root to tips, the flagged sequences were removed from the dataset. Application of this method leads to the elimination of 20 and 23 out of 333 sequences in the cases of tree T1 and T3. Altogether, T1, T2 and T3 have respectively 313, 333 and 310 tips (sequences). The RF (Robinson-Foulds) distance [31] of these tree topologies is shown in Table 1. The Newick format of all phylogenetic trees, which were used in downstream analyses, is given in Additional file 2.

**Table 1:** RF (Robinson-Foulds) distances between tree T1, T2 and T3

	T1	T2	T3
**T1**	0	233	220
**T2**	233	0	7
**T3**	220	7	0

RF is calculated using [51]

Finally, for the three topologies, the branches of the phylogenetic tree were divided into 4 conditions according to the host HLA types (see section Definition of the amino acid selective effects.)

### Model

#### Notations

We consider a coding sequence of length *N* (*N* being the number of coding positions, or equivalently *3N* is number of nucleotide sites). The number of conditions (e.g., HLA types) is defined by *K*. All the indices used in this paper conform to the following conventions:

- □ Codon positions (sites) *i є [1, N]*
- □ Conditions *k є [1, K]*
- □ Codon states *c є [1, 61]*
- □ Nucleotide states *n є* [1, 4]
- □Amino acid states *a є* [1, 19]

### Model of codon substitution

The rate of evolution by point substitution is the result of a complex interplay between mutation, selection and random drift. Drawing inspiration from previous developments in statistical molecular evolution [1, 2, 8, 9, 11], we modeled this process at the codon level, as a multiplicative combination of mutation rates and selective effects (the latter implicitly including the contribution from random drift).

The mutation process is assumed to be homogenous over time and along the sequence. It is modelled as a Markovian general time-reversible process, parameterized in terms of the relative exchange rates (*ρ*) between nucleotides and the stationary probability (equilibrium frequency) of the target nucleotide (*π*). Thus, the rate of substitution from nucleotide *n*_*1*_ to nucleotide *n*_*2*_ is equal to:

$$ {Q}_{n_1{n}_2}=\frac{1}{Z}{\uprho}_{n_1{n}_2}{\uppi}_{n_2} $$

Where, Z is the normalization factor:

$$ Z=\sum_{n_1}^{n_2}{\uprho}_{n_1{n}_2}{\uppi}_{n_2} $$

The set of relative exchangeabilities between nucleotides is constrained to be symmetric:

$$ {\uprho}_{n_1{n}_2}={\uprho}_{n_2{n}_1} $$

for all *n*_*1*_ *,n*_*2*_ *є [1 ,4]*

In addition, it is normalized:

$$ \sum_{n_1}^{n_2}{\uprho}_{n_1{n}_2}=1 $$

The vector *π* of equilibrium frequencies is also with the constraint

$$ \sum_n{\uppi}_n=1 $$

The selective forces, on the other hand, are both condition- and position-specific. The modulations across conditions and positions are mediated exclusively by the encoded amino acid sequence. Accordingly, for each position *i* and each condition *k*, we introduce an array of 20 non-negative fitness factors ${F}^{ik}=\left({F}_a^{ik}\right) a\in \left[1,20\right]$, one for each amino acid. In the following, these 20-dimensional vectors will be referred to as amino acid *fitness profiles*. Thus, we have distinct fitness profiles across positions, and for a given position, the fitness profile over the 20 amino acids is further modulated across conditions. How these fitness profiles are defined in practice is explained in more detail below (section; Definition of the amino acid selective effects).

Given a mutation matrix and a set of amino acid fitness profiles, we considered two alternative approaches for expressing substitution rates between codons as a function of the fitness of the amino acids. The first is a phenomenological approach, while the second is more mechanistic in its inspiration.

### Phenomenological model (M1)

The phenomenological model is similar, in its general form, to the models explored by Rodrigue et al. [8], or, in a slightly different parameterization, to the models considered in Robinson et al. [11]. Specifically, consider a given position *i* along the sequence, and a given condition *k* along the tree. Consider also two codons, *c*_*1*_ and *c*_*2*_, differing only at one position and with nucleotides *n*_*1*_ and *n*_*2*_ at that position. These two codons encode for amino acids *a*_*1*_ to *a*_*2*_, respectively. Then, the rate of substitution between these two codons is given by:

$$ {R}_{c_1{c}_2}^{ik}={Q}_{n_1{n}_2}\times \sqrt{\frac{F_{a_2}^{ik}}{F_{a_1}^{ik}}} $$

Thus, according to this model, the rate of substitution is proportional to the mutation rate, while being influenced by the selection operating at the amino acid level, through the fitness factors ${F}_a^{ik}$: the substitution rate is higher (resp. lower) than the neutral substitution rate if the fitness of the final amino acid is greater (resp. smaller) than the fitness of the initial amino acid. Note that, if the two codons are synonymous, i.e. if *a*_*1*_ *= a*_*2*_, then the substitution rate is simply equal to the mutation rate defined by the nucleotide transition matrix *Q*. Finally, the model considers only point substitutions, and therefore, the substitution rate is assumed to be equal to zero between codons differing at more than one nucleotide position. Thus, altogether:

$$ {R}_{c_1{c}_2}^{ik}=\left\{\begin{array}{l}{Q}_{n_1{n}_2}\kern4.1em \mathrm{Synonymous}\hfill \\ {}{Q}_{n_1{n}_2}\times \sqrt{\frac{F_{a_2}^{ik}}{F_{a_1}^{ik}}}\kern1em \mathrm{Non}\hbox{-} \mathrm{synonymous}\hfill \\ {}0\kern5.24em {\mathrm{c}}_1\mathrm{and}\ {\mathrm{c}}_2\mathrm{differ}\ \mathrm{at}\ \mathrm{more}\ \mathrm{than}\ \mathrm{one}\ \mathrm{site}\hfill \end{array}\right. $$

This formulation ensures that the average number of synonymous substitutions per unit length is equal to 1. Here, the selection factor modulates the rate of non-synonymous substitution.

### Mechanistic model (M2)

The second approach is inspired by a mechanistic argument based on first principles of population genetics, as initially suggested by Halpern and Bruno [9]. Consider again the substitution rate between codon *c*_*1*_ to *c*_*2*_ at site *i* and condition *k*. First, we define a scaled selection coefficient (scaled by effective population size *N*_*e*_), associated with codon *c*_*2*_, seen as a mutant in the context of a population in which the wild-type allele is *c*_*1*_. This scaled selection coefficient is given by:

$$ {S}_{a_1{a}_2}^{ik}=1\mathrm{n}\left(\frac{F_{a_2}^{ik}}{F_{a_1}^{ik}}\right) $$

Then, the rate of substitution between codon *c*_*1*_ and *c*_*2*_ is given by the product of the mutation rate and the relative fixation probability *P* (i.e. relative to neutral). This fixation probability is itself dependent on the scaled selection coefficient. Using the classical diffusion approximation, this relative fixation probability can be expressed as:

$$ {P}_{fix}=\frac{S_{a_1{a}_2}^{ik}}{1-{e}^{-{s}_{a_1{a}_2}^{ik}}} $$

So that the rate of substitution between codons is given by

$$ {R}_{c_1{c}_2}^{ik}=\left\{\begin{array}{l}{Q}_{n_1{n}_2}\kern6em \mathrm{Synonymous}\hfill \\ {}{Q}_{n_1{n}_2}\times \frac{S_{a_1{a}_2}^{ik}}{1-{e}^{-{S}_{a_1{a}_2}^{ik}}}\kern1em \mathrm{Non}\hbox{-} \mathrm{synonymous}\hfill \\ {}0\kern6.36em {\mathrm{c}}_1\;\mathrm{and}\;{\mathrm{c}}_2\;\mathrm{differ}\ \mathrm{at}\ \mathrm{more}\ \mathrm{than}\ \mathrm{one}\ \mathrm{site}\hfill \end{array}\right. $$

Again, we see that the rate of substitution is higher (resp. lower) than the neutral substitution rate if the non-synonymous mutation leads to an increase (resp. a decrease) in the fitness of the sequence.

### Definition of the amino acid selective effects

**Fig. 1:**
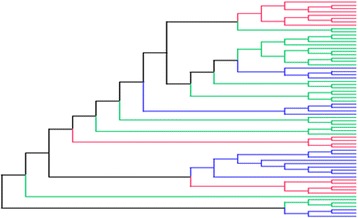
Illustrative phylogenetic tree of HIV-1 Gag sequences. Different colors along the tree show different selection regimes for the corresponding sequences. *Black branches* for between-patients, *green* for within-patients, *red* and *blue* for HLA-B57 and HLA-B35 categories, respectively. All tree topologies are such categorized

In principle, the amino acid fitness profiles associated with each site and each condition, ${F}_a^{ik}$, could be considered as independent arrays, both across sites and across conditions. However, most of the amino acid conservation (due to purifying selection) observed along the sequence is in fact condition-independent. Against this globally invariable fitness background, the modulations of the fitness landscape induced by condition-dependent effects (such as the HLA type of the host) are likely to be comparatively small. In this context, considering amino acid selective effects as totally independent random effects across conditions would imply that the invariable background would be re-estimated independently for each condition, potentially resulting in a loss of statistical power. Therefore, as a more powerful alternative, we explicitly defined amino acid selection in terms of a log-additive superposition of a global background and condition-dependent differential selective effects, as follows. First, a baseline or global fitness profile is defined for each position. That is, for position *i*, we define a 20-dimensional vector $\left({G}_a^i\right)$, for *a є [1, 20]*. This vector is drawn from a uniform Dirichlet distribution independently at each site. This baseline defines the fitness landscape under condition 0, which is therefore taken as our reference condition (black branches in Fig. 1).

Next, selection is modulated across conditions through the use of condition-specific differential selection profiles. Thus, for position *i* in condition *k*, we define a 20-dimensional vector $\left({D}_a^{ik}\right)$, for *a є [1, 20]*. Unlike the baseline profiles, which are positive (and sum to 1), those differential selection effects can be positive or negative. A positive (resp. negative) coefficient means that the fitness of the corresponding amino acid is increased (resp. decreased) in the target condition, compared to the reference condition. The differential selection profiles are drawn *iid* from a Normal distribution of mean 0 and condition-specific variance *σ*_*k*_^*2*^.

Altogether, the condition-specific fitness profiles are constructed as follows:

$$ \begin{array}{l}{F}_a^{i0}={G}_a^i\hfill \\ {}{F}_a^{i1}={G}_a^i{e}^{D_a^{i1}}\hfill \\ {}\begin{array}{l}{F}_a^{i k}={G}_a^i{e}^{\left({D}_a^{i1}+{D}_a^{i k}\right)}\\ {}\end{array}\hfill \\ {} k\in \left[2,,, K\right]\hfill \end{array} $$

Note that we have used a two-level system for introducing the differential effects (i.e. a different equation for *k = 1* and *k > 1*). This is motivated by the fact that we need to discriminate both among branches that are between hosts and within the same host, and among hosts with differing HLA backgrounds. Thus, it reflects the differential between within-host (*D* ^*i*1^) and between-host (*G* ^*i*^) selection regions, while representing specific selective features more specifically associated to differing HLA backgrounds (*D* ^*ik*^)_*kє [*2,*K]*_. In the case of HIV-1, we consider 2 focal HLA backgrounds (B57+ and B35+), against a default B57−/B35- background. Thus, we define a total of 4 different conditions (*K = 4*), and the branches of the tree are partitioned according to 4 different selection regimes (Fig. 1): first, we distinguished between the branches connecting the host-specific groups of sequences (between-patient condition) and the branches within each host-specific group of sequences (within-patient condition). Among the latter set of branches, we further distinguished among patients according to their HLA-type: either between HLA-B57+ and HLA-B57- patients, or between HLA-B35+ and HLA-B35- patients. The HLA-B57 type is known to be associated with the control of viremia [32, 33] whereas HLA-B35 is known as the HLA related to the fast progression of the disease [34, 35].

An important point should be emphasized concerning the statistical formalization of the fitness landscape and of its modulations across sites and across conditions. Conceptually, the arrays of global and condition-specific fitness effects should be considered, not as parameters, but as random-effects across sites, which are integrated over a distribution (respectively, a Dirichlet and a Normal distribution for the global and differential effects). This integration is done implicitly, through the MCMC sampling (see below). As a result, the aim of the model introduced here is not to achieve accurate and asymptotically consistent point estimation of site- and condition-specific fitness effects: in most cases, the information for inferring such fitness effects will be limited. Instead, it is to draw inference based on the complete posterior distribution. A more specific objective is to single out those relatively few cases for which there is sufficient information to infer, with high posterior probability, the presence of a differential selective effect between two conditions. One important desirable property of this type of inference is to allow for a reasonably good control of the fraction of false discoveries among those cases that are selected based on a high posterior probability of a differential effect. This is something which is investigated through posterior predictive simulations (see below).

### Priors

The topology (*τ*) of the tree is fixed. The parameters of the model consist of branch lengths, *l*_*j*_ (1 < *j* < 2*N*-3 where *N* is the number of sequences), nucleotide exchangeabilities *ρ* and nucleotide equilibrium frequencies *π*. The priors that we used are as follows: on branch lengths: a product of independent exponentials of mean *λ*; the hyperparameter *λ* is from an exponential distribution of mean 0.1; on relative exchangeability rate: a product of exponentials of mean 1; on mutational equilibrium frequency: a uniform Dirichlet distribution. As mentioned above, the site-specific fitness profiles (*G*) and differential fitness effects (*D*) are random-effects, integrated over Dirichlet and normal distributions, respectively.

### MCMC

We used Markov chain Mont Carlo (MCMC) to sample the parameters of the model from their joint posterior distribution. We used a graphical model environment previously introduced in [36], heavily relying on data augmentation and parameter expansions methods, such as described in particular in [37]. Briefly, a MCMC cycle consists of an alternation between two steps: first, a detailed substitution history at each coding site is Gibbs-sampled, from the posterior distribution conditional on the current parameter configuration. Second, conditional on these augmented data, the parameters and the random-effects across sites are updated through a large series of Metropolis-Hastings moves, cycling over all parameters or random variables of the model.

For the nucleotide equilibrium frequencies *π* and the global fitness profiles *G,* which are under the constraint that they should sum to 1, we used constrained move as explained in [36]. Branch lengths *l* and exchangeabilities *ρ*, which are positive real numbers, were updated using multiplicative moves [36]. Convergence of several key parameters and key sufficient statistics was monitored first by plotting their summary statistics as a function of number of iterations (points) for two independent runs; and second by using the *tracecomp* program (from the Phylobayes suite [38]) to compare the samples obtained under independent runs. *Tracecomp* gives an estimate of the discrepancy between the two runs, as well as the effective sample size, for several key parameters and statistics of interest. In the present case, the minimum effective size was greater than 300 and the discrepancy less than 0.2 for most statistics. After exclusion of the burn-in, posterior estimates were estimated by averaging over the remaining of the MCMC chain (approximately 1500 points for the empirical analyses, 1000 points for the simulations). As an additional control of the reproducibility of the MCMC analysis, we also checked that the posterior mean differential selection factors for all amino acids at all sites, as well as the associated posterior probabilities of a positive effect, were consistent between two independent runs (posterior probability correlation coefficient R^2^ > 83% in all cases, see Additional file 3: Figure S1 and Additional file 4: Figure S2).

### Simulations

Simulations were conducted using a modified version of the posterior predictive formalism [39, 40]. In all cases, parameter configurations were drawn from the posterior distribution under the 4-condition model fitted on the HIV dataset. Then, in a first series of simulations, the differential selection effects across differential conditions were set to 0, while the global selection profiles were left unchanged, thus giving empirically calibrated simulation replicates under the null hypothesis of no differential effect across conditions. These simulations were conducted to estimate the rate of false positives.

In a second series of simulations, we implemented a sparse distribution of differential selection effects across sites, with various fractions (f = 0.5, 0.1 and 0.05) of sites with non-zero effects. Sites with non-zero effects were chosen uniformly at random, independently for conditions 2 (HLA B57+) and 3 (HLA B35+), and were endowed with differential condition effects independently drawn from a reflected gamma distribution of mean 1 and shape parameter 2. This second series of simulations was conducted to evaluate the precision and sensitivity of the method. In both cases, the phenomenological (M1) and the mechanistic (M2) models were investigated, and simulations were conducted based on 10 parameter configurations sampled from the posterior distribution (10 points regularly spaced from the MCMC run), yielding a total of 10 replicates per condition.

For all simulations, the full model (with *K = 4* conditions) was then applied to these simulated data. For a given pair of condition (e.g., HLAB57+ versus HLAB57-), and for several *α* levels, the number of positions inferred to be under differential selection with posterior probability greater than *1-α* was determined. In the context of the first series of simulations (no differential selection simulated), dividing this number by the total number of positions times the number of amino acids gives the rate of false positives, which was tabulated for several values of *α*. For the second series of simulations (with differential selection simulated), the discoveries made at a given threshold were compared with the true differential selection values, and the precision (fraction of true discoveries over all discoveries) and the sensitivity (fraction of true discoveries over all differentially selected sites) were determined as a function of the significance threshold. A discovery is deemed true if the true differential selection effect is non-zero and of the same sign as the inferred differential selection effect.

## Results

### Simulation analyses

The properties of the model were first investigated through simulations. Since the main application of the model introduced here is to identify positions for which specific amino acids are under differential condition-dependent selection pressure, the simulation analyses were more specifically designed to evaluate the rate of false positives of the method, as well as its precision and sensitivity. In order to ensure that the conclusions of the simulations are relevant to the empirical situations considered here, simulations were calibrated against parameter estimates obtained from the empirical analyses on the HIV dataset. This was done using a modified version of the posterior predictive formalism [39, 40].

A first series of 10 replicates were produced under the null model assuming no differential selection effect across conditions — thus, considering a constant fitness landscape over the whole phylogenetic tree. The model with *K = 4* conditions was then applied to these simulated data. For a given pair of condition (e.g., HLAB57+ versus HLAB57-), and for different α levels, the number of positions inferred to be under differential selection with posterior probability greater than *1-α* was determined, giving us an estimate of the false positive rate as a function of the stringency of the selection. As can be seen from Table 2, for reasonable posterior probability thresholds, the rate of false positive is low, on average, reaching 5% for *1-α* = 0.65, and lower than 1% for *1-α* > 0.8.

**Table 2:** False Positive Rates (FPR) for different conditions and posterior probability thresholds under model M1 and M2

	M1		M2	
Threshold	Mean number of FP	FPR	Mean number of FP	FPR
Condition 1 (within-patients)				
>0.55	1843.7	20.8	1845.8	20.8
>0.60	1112.7	12.6	1166.8	13.2
>0.65	684.9	7.7	737.8	8.3
>0.70	316.9	3.6	334.5	3.8
>0.75	173.1	2.0	181.8	2.1
>0.80	81.8	0.9	86.5	1.0
>0.85	26.0	0.3	31.7	0.4
>0.90	7.1	0.1	4.6	0.1
>0.95	0.75	0.01	0.1	0.0
Condition 2 (HLA-B57+)				
>0.55	1004.1	11.3	957	10.8
>0.60	456.3	5.15	471	5.3
>0.65	229	2.58	237.1	2.7
>0.70	88.9	1	78.7	0.9
>0.75	31.3	0.3	27.3	0.3
>0.80	12.9	0.15	8.4	0.1
>0.85	3.8	0.04	1.6	0.02
>0.90	0.4	0	0.05	0
>0.95	0	0	0	0
Condition 3 (HLA-B35+)				
>0.55	1245.1	14	1226.5	13.8
>0.60	632.4	7.1	683	7.7
>0.65	345.6	3.9	385.3	4.3
>0.70	141.4	1.6	148.1	1.7
>0.75	58.6	0.7	64.4	0.73
>0.80	25.3	0.3	23.1	0.3
>0.85	7.7	0.1	6.5	0.07
>0.90	1.2	0.01	0.9	0.01
>0.95	0	0	0	0

This simulation experiment illustrates a point about the Bayesian approach used here: using Normal distribution centered on 0 enforces shrinkage of the differential fitness effects across positions towards 0 (i.e. the model is centered on the null hypothesis representing an absence of selective difference between conditions). One important consequence of this choice is that, in the absence of a sufficiently strong empirical signal able to counteract this prior, the method will typically not infer high posterior probability support for differential selective effects. Note that these simulations, which have been calibrated against the empirical dataset of interest, can also be used to obtain a rough estimate of the fraction of false discoveries, by comparing, for a given threshold, the total number of discoveries (d) on the real dataset with the mean number of false positives (d0) under the simulations. An estimate of the fraction of false discoveries is then given by d0/d (see below).

A second series of simulations was conducted, assuming the presence of modulations of the fitness landscape across conditions, with various fractions of sites under non-zero differential selection effects. For a given pair of condition (e.g., HLAB57+ versus HLAB57-), and for a given *α* level, the set of discoveries at level *α* (i.e. the set of all positions/amino acid pairs such that the posterior probability of a differential selection effect between the two conditions is greater than *1-α*) was determined. A discovery was then deemed to be false if the true selective effect for that amino acid at that position is either 0 or of the opposite direction. The precision and sensitivity were tabulated as a function of 1-α (Tables 3 and 4, for condition 2 and 3, respectively).

**Table 3:** Precision (prec) and sensitivity (sens) as a function of the proportion of differentially selected sites (f) in condition 2, under model M1 and M2

Threshold	M1						M2					
	f = 0.5		f = 0.1		f = 0.05		f = 0.5		f = 0.1		f = 0.05	
	Prec	Sens	Prec	Sens	Prec	Sens	Prec	Sens	Prec	Sens	Prec	Sens
>0.50	26.7	53.5	4.7	47.0	2.8	56.4	2.5	49.7	5.3	53.0	25.5	51.1
>0.55	39.1	9.0	7.7	7.6	4.7	9.3	4.0	8.4	8.3	8.4	37.5	8.5
>0.60	47.0	6.1	10.0	4.9	6.3	6.0	5.6	5.4	10.9	5.3	45.2	5.2
>0.65	55.2	4.3	14.1	3.5	9.0	4.4	8.2	3.8	14.3	3.4	52.8	3.4
>0.70	67.0	2.9	21.9	2.4	15.8	3.2	12.1	2.1	21.3	1.8	63.8	1.9
>0.75	78.7	2.0	34.4	1.8	25.7	2.5	23.3	1.4	33.0	1.1	78.2	1.1
>0.80	84.1	1.5	50.8	1.4	35.2	1.8	33.0	0.7	44.8	0.6	86.5	0.7
>0.85	91.2	1.1	66.2	1.0	49.0	1.2	41.4	0.3	73.7	0.3	92.7	0.3
>0.90	93.5	0.8	81.7	0.7	68.3	0.6	33.3	0.1	90.9	0.1	100	0.1
>0.95	96.0	0.4	93.3	0.3	86.7	0.3	0.0	0.0	100	0.01	100	0.03

**Table 4:** Precision (prec) and sensitivity (sens) as a function of the proportion of differentially selected sites (f) in condition 3, under model M1 and M2

Threshold	M1						M2					
	f = 0.5		f = 0.1		f = 0.05		f = 0.5		f = 0.1		f = 0.05	
	Prec	Sens	Prec	Sens	Prec	Sens	Prec	Sens	Prec	Sens	Prec	Sens
>0.50	26.6	53.2	5.2	51.6	2.5	49.8	2.7	54.3	5.1	51.0	26.1	52.2
>0.55	40.2	12.0	8.3	10.7	4.0	10.2	4.4	11.7	8.3	10.8	37.2	10.9
>0.60	47.2	8.3	10.7	7.6	5.5	7.4	5.7	7.9	10.5	7.6	44.1	7.4
>0.65	54.1	6.2	13.6	5.5	7.5	5.6	6.7	5.1	13.5	5.4	49.5	5.0
>0.70	64.7	3.9	21.4	3.7	11.9	3.6	9.9	2.9	21.4	3.3	60.2	2.9
>0.75	75.6	3.0	32.1	2.9	18.1	2.6	14.3	1.8	34.4	2.5	70.7	1.9
>0.80	83.5	2.4	44.4	2.3	27.5	2.1	22.7	1.2	50.3	1.8	80.8	1.2
>0.85	90.7	1.8	61.6	1.8	46.4	1.8	38.2	0.8	67.1	1.1	88.7	0.7
>0.90	93.7	1.4	77.3	1.3	64.1	1.3	66.7	0.4	79.4	0.6	97.1	0.4
>0.95	96.8	1.0	89.2	0.8	89.1	0.9	100	0.1	100	0.2	98.3	0.1

As we see in Tables 3 and 4, for a given posterior probability threshold, the precision decreases when the proportion of differentially selected sites (f) decreases. This reflects the fact that the number of true positives is directly proportional to the proportion of sites with differential selection effects, while the number of false positives remains stable. Overall, the power of the method is relatively low. Under a precision of 0.9 (10% of false discoveries), the sensitivity (or recall) is between 1 and 0.1%, depending on the model and the exact simulation condition (i.e. less than 1% of the differentially selected positions are detected).

### Analyses of HIV empirical data

We applied our DS model to a dataset of HIV coding sequences (encoding the Gag protein) obtained from 41 patients. We used this dataset for two reasons. First, it contains multiple sequences for each patient, thus providing empirical information about within-host evolution of viral genetic sequences. Second, the HLA type of the patients is known, and therefore, it is possible to correlate the amino acid patterns observed in viral sequences with the HLA type of the host.

**Fig. 2:**
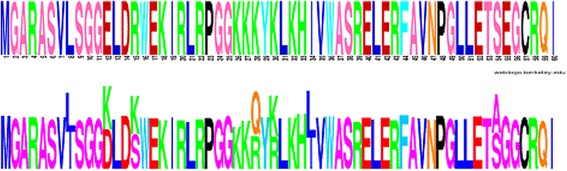
Comparison of global selection profile estimated by DS model (bottom) with HIV reference sequence HXB2 (top). The first 60 amino acids are shown. The reference logo was made using Weblogo [52]

Accordingly, in this study, we partitioned the phylogenetic tree relating the viral sequences into different categories. A global reference selection profile was estimated by our method. This reference fitness landscapes, which captures the baseline site-specific amino acid preferences in the form of site-specific vectors of 20 fitness factors (one for each amino acid), can be visualized using a graphical logo representation [41] and compared with the reference HIV-1 sequence (HXB2, the first 60 coding positions are shown in Fig. 2). The selection profile inferred with our method is highly similar to the reference sequence (the fittest amino acid corresponds to the amino acid of the reference sequence at 86% of the coding positions). In some cases, compared to the reference sequence, the fitness profile suggests a distinct but biochemically similar dominant amino acid (e.g., position 15, K instead of R), or several equally fit amino acids at one position (e.g., position 30, K and R). This corresponds to the actual sequence variation observed in our empirical alignment. Altogether, this global reference selection profile illustrates that HIV evolution occurs on a background characterized by strong purifying selection, allowing for a very limited set of amino acid sequences for the viral protein.

**Fig. 3:**
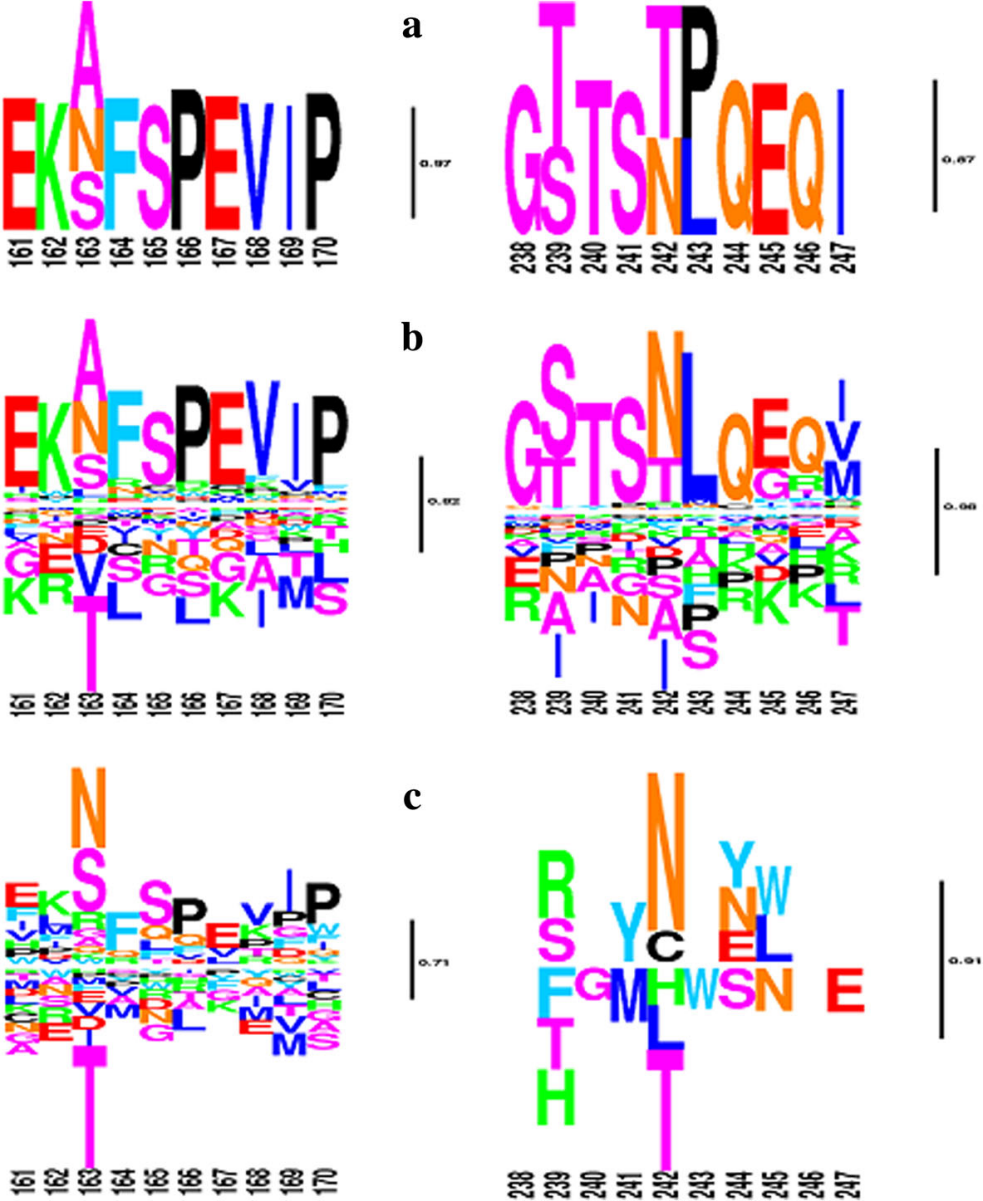
Global and differential selection profiles (for HLA-B57). (**a**) Global selection profile (G). (**b**) Differential selection profile contrasting between- and within-patient selection. (**c**) Differential selection profile for HLA-B57+ versus HLA-B57-. The posterior probability (pp) of an increased fitness for N and a decreased fitness for T at position 242 (TW10 epitope), in HLA-B57+ compared to HLA-B57-, is 0.93 and 0.87, respectively. At position 163 (KF11 epitope), the fitness of N is increased with a pp. of 0.77. The logos are filtered for pp. below 0.05. Heights are proportional to posterior mean differential selective effects

**Fig. 4:**
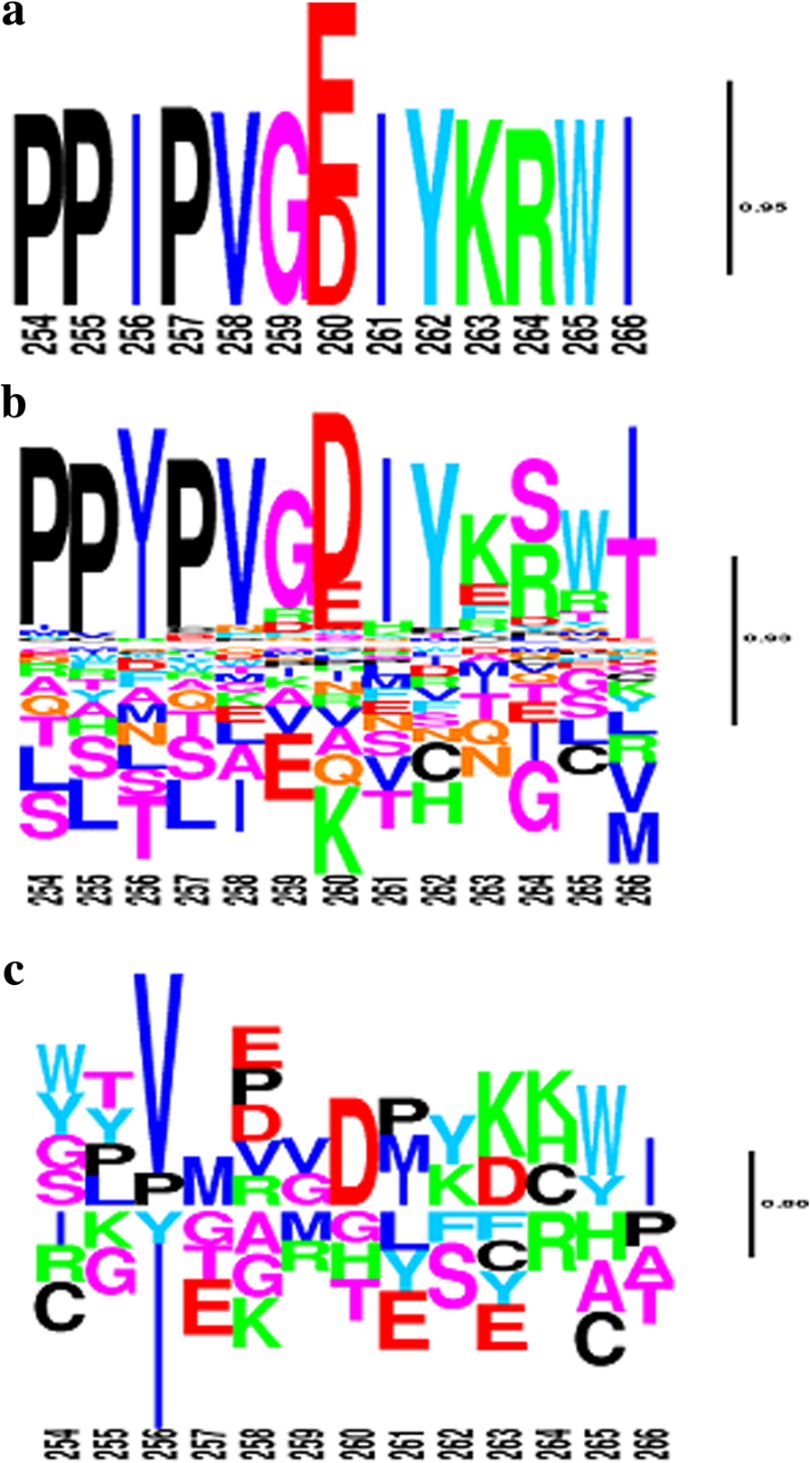
Global (**a**) and differential selection profiles, contrasting within and between patients (**b**) and HLA-B35+ versus HLA-B35- (**c**). In **c**, the posterior probability (pp) of fitness shift from D to E at position 260 is 0.81. The logos are filtered for pp. less than 0.05. Heights are proportional to posterior mean differential selective effects

**Fig. 5:**
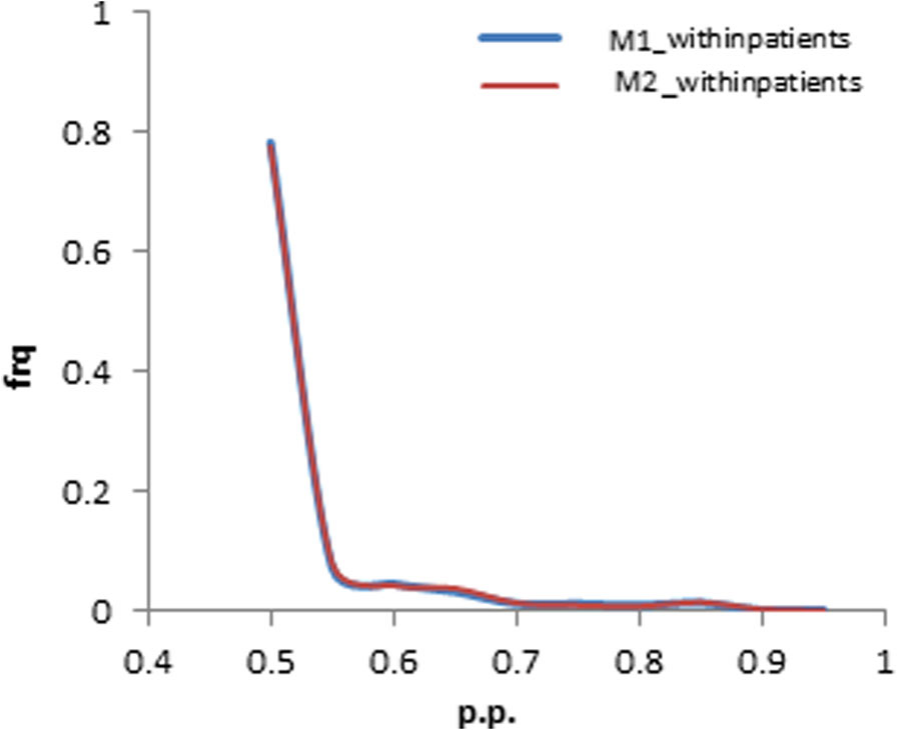
Posterior probability frequency plots of differential selection effects across all amino acid-positions; phenomenological (M1) vs mechanistic (M2). Posterior probability of the majority of amino acid-position lies between 0.5–0.6

Against this background fitness landscape, our model then estimates differential selection profiles between each pair of conditions: first, between within-host and between-host (Figs. 3-b and 4-b), and second, among within-host sequences, between HLA-B57- and HLA-B57+ sequences (Fig. 3-c), or between HLA-B35- and HLA-B35+ sequences (Fig. 4-c). The logos represented on Figs. 3 and 4 indicate whether the fitness of any particular amino acid is inferred to be increased (above the line) or decreased (below the line) with posterior probability >0.80, at a given position, between the two conditions being compared. These figures only give point estimates for the differential effects. In practice, the posterior probability support associated to these estimates is most often low, at about 0.5 (Fig. 5), except for a small subset of positions for which stronger evidence for a differential selection effect is inferred by the model. These more clear-cut cases represent our findings, which are given in Table 5 for the two model settings. In the following, we report the findings for two thresholds, at 0.80 and 0.90. We will refer to the corresponding discoveries as weakly and strongly supported findings, respectively.

**Table 5:** Number of differentially selected amino acid-positions with posterior probability >0.80 and >0.90, in different conditions under model M1 and M2

Threshold	Model	Within-patient	B57^+^ patients	B35^+^ patients
>0.80	M1	281	15	48
>0.80	M2	286	5	30
>0.90	M1	54	2	13
>0.90	M2	56	0	1

By far, we observe in Table 5 that the largest number of differentially selected amino acid variants is found when comparing the within- and between-patient conditions, with more than 280 findings under both models. On the other hand, the corresponding profiles suggest that this is mostly due to a global difference intact the intensity of selection (or a global difference in statistical power), rather than to specific selective differences between the two conditions (see Discussion).

The differences between alternative HLA backgrounds, on the contrary, seem to be more specific. Comparing the number of findings reported in Table 5 under conditions 2 and 3 with the mean number of false positives in simulation experiments under the null model with no differential selection and for the same threshold (Table 2) gives a rough estimate of the fraction of false discoveries. Thus, for a threshold of 0.9, the fraction of false discoveries is approximately 20% in condition 1 and 9% in condition 3 under model M1, whereas model M2 does not seem to lead to a significant enrichment compared to the expected number of false positives. Therefore, in the following, we consider only model M1.

The findings under model M1 are listed in more details (position, amino acid, lower and upper 95% credible intervals and posterior probability support) in Tables 6 and 7 for B57+ and B35+ conditions, respectively. For each finding, the direction of the effect (whether the fitness is increased or decreased between the two conditions being tested) is indicated, together with the posterior probability that the effect is >0 or <0 (depending on the direction of the effect). Among our findings, there are some known mutations identified in association with specific HLAs. Two important HIV-1 escape mutations defined in B57+ patients are T242 N and A163X in epitopes TW10 [42, 43] and KF11 [44, 45], respectively. X at position 163 is mostly P and N. The logos of the corresponding regions are shown in Fig. 3. The selection factors estimated at these positions are in agreement with these previously known escape mutations.

**Table 6:** List of differentially selected amino acids for B57+ hosts with posterior probability > 0.80

Position	Amino acid	Posterior probability	Median	Lower	Upper	Fitness
242	N	0.93	1.36	-0.37	3.07	Increased
248	G	0.91	-1.20	-2.82	0.45	Decreased
30	Q	0.89	1.09	-0.69	2.92	Increased
242	T	0.87	-0.95	-2.55	0.78	Decreased
30	K	0.87	-0.96	-2.49	0.69	Decreased
357	A	0.86	0.94	-0.73	2.86	Increased
15	R	0.86	0.72	-1.01	2.41	Increased
118	A	0.85	-0.93	-2.69	0.79	Decreased
239	S	0.85	1.02	-0.95	2.64	Increased
137	L	0.82	-0.86	-2.55	0.93	Decreased
326	S	0.81	0.79	-1.28	2.46	Increased
357	G	0.81	-0.78	-2.55	0.97	Decreased
280	T	0.80	0.83	-0.79	2.43	Increased
12	E	0.80	0.71	-0.96	2.43	Increased
248	E	0.80	0.66	-0.97	2.42	Increased
223	I	0.80	-0.70	-2.28	1.02	Decreased

The amino acid-positions are sorted according to the posterior probability score. Median, lower and upper 95% credible intervals and the direction of the effect on fitness (increased or decrease) are indicated

**Table 7:** List of differentially selected amino acids for B35+ hosts with posterior probability >0.80

Position	Amino acid	Posterior probability	Median	Lower	Upper	Fitness
46	L	0.97	1.69	-0.05	3.44	Increased
34	L	0.96	1.52	-0.31	3.19	Increased
252	H	0.96	1.59	-0.18	3.28	Increased
111	S	0.93	-1.15	-2.72	0.49	Decreased
127	Q	0.93	-1.11	-2.74	0.48	Decreased
376	V	0.93	1.16	-0.49	2.68	Increased
312	D	0.92	1.23	-0.55	3.06	Increased
137	M	0.92	1.26	-0.47	3.22	Increased
252	N	0.92	-1.05	-2.60	0.48	Decreased
30	K	0.92	-1.05	-2.44	0.52	Decreased
248	A	0.91	1.25	-0.41	3.07	Increased
310	T	0.91	1.25	-0.54	2.97	Increased
441	H	0.89	0.95	-0.43	2.46	Increased
46	V	0.89	-1.06	-2.74	0.52	Decreased
67	A	0.89	1.09	-0.66	2.82	Increased
111	C	0.88	1.08	-0.75	2.76	Increased
375	V	0.88	-0.85	-2.48	0.72	Decreased
255	V	0.88	1.08	-0.79	2.61	Increased
441	Y	0.87	-0.92	-2.37	0.53	Decreased
405	I	0.86	0.94	-0.72	2.51	Increased
15	Q	0.86	0.94	-0.77	2.84	Increased
138	L	0.86	-0.90	-2.41	0.76	Decreased
376	I	0.85	-0.81	-2.26	0.67	Decreased
127	T	0.85	1.01	-0.86	2.83	Increased
69	Q	0.84	-0.79	-2.37	0.78	Decreased
81	A	0.84	0.94	-0.74	2.65	Increased
176	A	0.84	0.86	-0.88	2.86	Increased
280	T	0.83	0.96	-0.86	2.40	Increased
348	S	0.83	0.97	-0.90	2.87	Increased
61	I	0.83	0.77	-1.14	2.61	Increased
81	T	0.83	-0.82	-2.41	0.85	Decreased
268	M	0.82	0.81	-0.81	2.45	Increased
280	A	0.82	-0.82	-2.41	0.85	Decreased
388	K	0.82	0.74	-0.90	2.37	Increased
389	P	0.82	0.81	-0.81	2.45	Increased
397	R	0.82	0.72	-1.00	2.53	Increased
95	R	0.82	0.77	-0.83	2.39	Increased
68	I	0.81	0.87	-1.14	2.67	Increased
215	L	0.81	-0.73	-2.19	0.70	Decreased
118	T	0.81	0.70	-0.95	2.33	Increased
260	D	0.81	0.75	-1.00	2.48	Increased
54	A	0.81	0.75	-0.96	2.52	Increased
93	A	0.80	0.73	-1.04	2.44	Increased
28	K	0.80	-0.66	-2.46	1.06	Decreased
58	K	0.80	0.69	-1.31	2.34	Increased

The amino acid-positions are sorted according to the posterior probability score. Median, lower and upper 95% credible intervals and the direction of the effect on fitness (increased or decrease) are indicated

Intriguingly, the T/N escape variant at position 242 (TW10 epitope) is not recovered by the mechanistic model (M2), suggesting that the phenomenological model is more adequate to predict differential selection patterns. This confirms our simulation studies, proving that the phenomenological model has a greater detection power. Also of interest, our method does not infer that T is preferred in a B57- environment, whereas N is favored in a B57+ background. Instead, it suggests that both amino acids are acceptable in a B57- environment, but that N becomes the only one favored in B57+ patients. A similar pattern is observed for the A163X escape mutation, with posterior probability = 0.77. One known mutation for B35+ individuals is E260D in NY10 epitope [46]. Our method detects this mutation to be under condition-specific selection with posterior probability of 0.81 (Fig. 4).

### Robustness to the choice of the tree topology

The method relies on a fixed tree topology. However, in practice, the tree is reconstructed with errors. To test the robustness of the inference, we conducted the analysis under three alternative tree topologies, under the M1 model). We refer to these trees as tree T1, T2 and T3 (see methods). The set of differentially selected positions were found to be very similar for all trees (Table 8), suggesting that the exact details of the tree topology are not so important in the present context.

**Table 8:** Number of differentially selected amino acid-positions with posterior probability >0.80 and >0.90 obtained by M1-DS model using tree T1, T2 and T3

Threshold	Tree topology	B57+ patients	B35+ patients
>0.80	T1	15	48
>0.80	T2	12	51
>0.80	T3	18	48
>0.90	T1	2	13
>0.90	T2	2	10
>0.90	T3	3	12

**Fig. 6:**
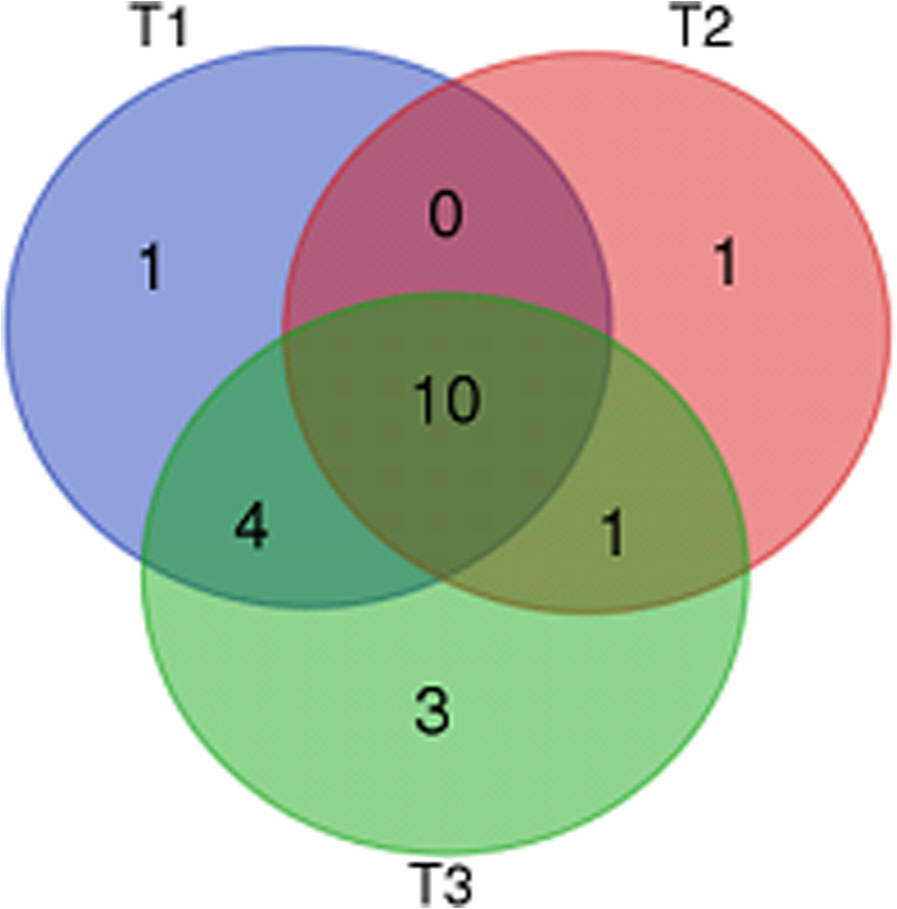
Venn diagram of positions found with posterior probability >0.80, using tree T1 (NJ topology), T2 (MrBayes topology with constraint) and T3 (MrBayes topology without constraint). 10 positions are shared by three topologies

By comparing the number of positions declared significant for each threshold (shown in Table 8), we see that for B57+ condition, the number of findings is very close in different tree topologies (15, 12 and 18 under posterior probability >0.80, and 2, 2 and 3 under posterior probability >0.90). We also summarized the common positions between the three topologies as a Venn diagram in Fig. 6. There is only one position in T1 which is not recovered by T2 or T3. The majority of positions (10) were found by all trees. None of the discrepancies between analyses under differing topologies belong to the positions previously known to correspond to viral escape mutants. Altogether, the relatively small number of sequences that had to be removed, combined with the relative robustness of our result to the choice of the tree topologies despite their distances (specially between tree T1 and tree T2 and T3, see Table 1), suggests that the problems of multiple infection patterns, or tree reconstruction errors, have a globally marginal impact on our analysis.

## Discussion

Here, we have introduced a hierarchical Bayesian method for detecting adaptive patterns in protein-coding sequences as a function of known selective backgrounds. Compared with previously introduced methods [23, 24], our approach has several additional features. The approach of Carlson et al. [23], relying on a Bayesian network representation, is formulated at the codon level. In addition, it can accommodate epistatic effects (see introduction). Nevertheless, it is focused on the terminal branches of the phylogeny and therefore ignores potentially relevant empirical information from the deeper parts of the phylogenetic tree. The approach of Tamuri et al. [24–26], in contrast, fully integrates the empirical signal over the entire tree, and is thus much more similar, in spirit, to the present method. The main difference is in the statistical framework used to deal with site-specific effects (empirical Bayes versus maximum-likelihood estimation). The fact that our method integrates the empirical signal about more ancient codon substitutions opens interesting possibilities, in particular, for comparing short-term (within-host) and long-term (between-host) adaptive patterns. As it stands, however, the selection profiles obtained for between- and within-host are not yet so assuring: the within-host differential selection profiles obtained through our method (Figs. 3-b and 4-b) seem to partially reproduce the condition-independent amino acid fitness profiles (Figs. 3-a and 4-a). The reasons for such a redundant output are not totally clear. Deleterious mutations segregating within-host, but purified away in the long-term (and therefore absent from the deeper branches of the phylogeny connecting host-specific clusters) are an important difference between within- and between-host conditions. However, such segregating polymorphisms would be expected to result in an opposite pattern, leading to artefactual high selection coefficients in the within-host condition for unfit amino acids that are not observed in the between-host selection profiles. One alternative explanation for the observed redundancy would be that the law of condition-independent selection profiles across sites is not correctly captured by a Dirichlet distribution. Possibly for that reason, the remaining part of the condition-independent selective effects may be captured by the differential selection profile of the within-host condition. Ultimately, more sophisticated hierarchical Bayesian settings could be used, such as non-parametric priors [8]. The combination of condition- and site-specific effects is computationally challenging, and further algorithmic work is therefore needed in this direction to fully accommodate arbitrary distributions of random-effects across positions and conditions.

The distribution of differential selective effects across sites and conditions may also need additional statistical and computational developments in the long term. Here, we have used Normal distributions centered on 0 to model differential selective effects. Doing this leads to efficient soft shrinkage toward 0. However, this approach does not implement sparsity. All amino acids, at all positions and under all conditions, have non-zero differential selective effects with a posterior probability of one. Ultimately, sparse differential selection profiles (with only a small number of positions and amino acids displaying significant non-null differential selective effects) could be obtained through the use a spike-and-slab mixture model [47]. In this context, estimating the proportion of non-null effects, as well as the effect size distribution directly on the empirical data would have several advantages, including an increased power, more accurate quantification of the effect sizes, as well as a more direct control of the rate of false discovery. In addition, this hierarchical model would allow for testing the null hypothesis that the gene has no differentially selected positions, by simply comparing the full model with the one constrained so as to have a null proportion of differential effects.

As suggested by our simulation experiments, modelling differential selection effects as random variables, with a distribution centered on 0, ensures good regularity properties of the approach. On the other hand, the power of the approach appears to be rather low. Further development of the current approach, along the lines just suggested, combined with a more systematic comparison with the currently existing alternatives [23–26], will have to be conducted, in order to establish whether this low power is a specific weakness of the present method (in particular because of the lack of sparsity of the model), or more fundamentally an inherent limitation of the problem of detecting weak effects across a large number of coding sites and for all possible amino acids.

Two alternative models of the rate of change between codons were considered in this study: one purely phenomenological [8, 11], and another one that has a better mechanistic justification, based on first principles of population genetics. When applied to HIV sequences, the mechanistic model does not seem to lead to better results, compared to the phenomenological approach. In particular, it fails to detect known HLA-restricted escape mutations. The mechanistic model, however, makes several assumptions that are clearly not warranted in the present context: low-mutation approximation, and more fundamentally, a mutation-fixation paradigm [9, 48], which amounts to ignoring clonal interference. In sharp contrast, viral sequences evolve under a very high mutation rate, leading to strong clonal interference. Another consequence of the very high mutation rate is that segregating deleterious polymorphisms are expected to be present at a substantial frequency, something which is not correctly captured by the mutation-selection model: fundamentally, this model is meant to be applied to inter-specific data. Here in contrast, a meta-population model would be more adequate. The theoretical and computational developments in this direction still appear to be challenging.

Our method does not take into account epistatic interactions between positions. Yet, those interactions seem to play an important role in HIV evolution, in particular concerning escape mutations. Most escape mutations cause a viral fitness cost which leads to decreased replication of the virus [42]. Position 242 is under the strongest selection pressure from the immune system which corresponds to the ability of B57+ hosts to control the disease. T242 N mutation in B57+ individuals reverts in viruses transmitted to a HLA-mismatched host [43], which confirms that the mutation has a strong fitness cost for the virus in terms of replication capacity [49]. This fitness cost might be compensated for, to some extent, by mutations at other positions, mostly around the escape mutation. In sequences with T242 N mutation, the compensatory mutations H219Q, I223V, M228I/V, G248A and N252H has been identified [42, 43]. It has been reported that these mutations are significantly more frequent in HLA-B57+ patients with a progressing disease compared to HLA-B57+ non-progressors [42]. Here, we did not see significant differences for final amino acids (Q, V, I/V, A and H) between B57+ and B57- patients at those suppressing positions (their posterior probability is less than 0.70), although initial amino acids are strongly unfavored (posterior probability =0.80, 0.91, 0.77 for I, G and N at positions 223, 248 and 252, respectively). There may be two reasons for that; first, our model takes each site into account independently and codon co-variation is not considered. Secondly, contrary to escape mutations which revert in the HLA mismatch host, compensatory mutations do not tend to revert after transmission to HLA mismatch individuals [43]. For example, H219Q, the associated mutation to T242 N, is reported to be maintained after transmission from B57+ to B57- hosts. So, this mutation might be stable and spread in the population. As it stands, explicitly implementing epistatic effects in the context of the present modeling framework appears to be challenging, although not impossible [50].

## Conclusions

We proposed a phylogenetic differential selection model, which is able to find adaptive patterns in coding sequences influenced by selective environments. Applying the model to HIV-1 *Gag* sequences, leads to the detection of a few amino acid-positions that are differentially selected under different host HLA types, as HIV escapes from immune system through its fast evolution. The model is thus able to find known HLA-restricted mutations, as well as some new mutations, to be under differential selection. The power of our model is that it is capable of detecting both positive and negative selection pressure on each amino acid at each position under each environmental condition.

This DS model can be used in other situations in which differential selective effects are suspected, as a function of known predictors, for viruses (e.g., finding adaptive patterns of HIV sequences under the selection pressure of immune system or antiviral therapy provides an insight of the direction of HIV-1 evolution in different hosts with different genetic characteristics), or in other species (e.g., convergent adaptations of multiple lineages of plants, or animals, to specific environmental conditions (Parto S, Lartillot N: Molecular adaptation in Rubisco: discriminating between convergent evolution and positive selection using mechanistic and classical codon models, in preparation).

## Additional files

Additional file 1: **Table S1.** Dataset of 333 HIV-1 sequences from gag region. (DOCX 48 kb)

Additional file 2: **Table S2.** Newick format of tree T1, T2 and T3. (DOCX 18 kb)

Additional file 3: **Figure S1.** Posterior mean differential selection factors for all amino acids at all sites for two independent runs, for within-patients (a), B57+ patients (b) and B35+ patients (c). The correlation coefficient R^2^ is provided for each plot. (DOCX 112 kb)

Additional file 4: **Figure S2.** Posterior probability correlation for all amino acids at all sites for two independent runs, for within-patients (a), B57+ patients (b) and B35+ patients (c). The correlation coefficient R^2^ is provided for each plot. (DOCX 171 kb)

## Abbreviations

CTL: Cytotoxic T lymphocyte
DS: Differential selection
HLA: Human leukocyte antigen
LANL: Los Alamos National Laboratory
MCMC: Markov Chain Monte Carlo
