# Supplementary material for: Detecting consistent patterns of directional adaptation using differential selection codon models
Source: BMC Evol Biol. 2017 Jun 23;17:147. doi: 10.1186/s12862-017-0979-y (PMC5481935; doi:10.1186/s12862-017-0979-y)
Supplement: Supplementary file 1 — Table S1. Dataset of 333 HIV-1 sequences from gag region. (DOCX 48 kb) [file 12862_2017_979_MOESM1_ESM.docx]

Table S 1. Dataset of 333 HIV-1 sequences from gag region.

| **Accesion number** | **Patient ID** | **Subtype** | **HLA type** | **Country** |
| --- | --- | --- | --- | --- |
| AY331282 | 27177 | B | A*0202 A*0301 B*0702 B*1516 | US |
| AY331283 | 27177 | B | A*0202 A*0301 B*0702 B*1516 | US |
| AY331284 | 4574 | B | A*2601 A*6802 B*1510 B*1510 | US |
| AY331285 | 13621 | B | A*6601 A*6802 B*4201 B*5301 | US |
| AY331286 | 13621 | B | A*6601 A*6802 B*4201 B*5301 | US |
| AY331287 | 4575 | B | A*2301 A*3001 B*4201 B*5301 | US |
| AY331289 | 13509 | B | A*6801 A*7401 B*1503 B*5802 | US |
| AY331290 | 13509 | B | A*6801 A*7401 B*1503 B*5802 | US |
| AY331293 | 13227 | B | A*0301 A*2301 B*0702 B*1503 | US |
| AY331296 | 27178 | B | A*0308 A*3001 B*0705 B*4501 | US |
| AY331297 | 27178 | B | A*0308 A*3001 B*0705 B*4501 | US |
| AY332236 | 4574 | B | A*2601 A*6802 B*1510 B*1510 | US |
| AY423381 | 12939 | B | A3 A36 B15 B51 Cw3 Cw6 DR4 DR8 DQ7 | NL |
| AY423382 | 12939 | B | A3 A36 B15 B51 Cw3 Cw6 DR4 DR8 DQ7 | NL |
| AY423384 | 12939 | B | A3 A36 B15 B51 Cw3 Cw6 DR4 DR8 DQ7 | NL |
| AY423385 | 12939 | B | A3 A36 B15 B51 Cw3 Cw6 DR4 DR8 DQ7 | NL |
| AY423386 | 12939 | B | A3 A36 B15 B51 Cw3 Cw6 DR4 DR8 DQ7 | NL |
| AY779550 | 9869 | B | A2 A3 B57 B65 | CA |
| AY779551 | 9869 | B | A2 A3 B57 B65 | CA |
| AY779552 | 9869 | B | A2 A3 B57 B65 | CA |
| AY779553 | 6944 | B | A2 A11 B56 B62 Cw1 | CA |
| AY779554 | 6944 | B | A2 A11 B56 B62 Cw1 | CA |
| AY779555 | 6944 | B | A2 A11 B56 B62 Cw1 | CA |
| AY779557 | 13458 | B | A2 A24 B7 B13 | CA |
| AY779558 | 13458 | B | A2 A24 B7 B13 | CA |
| AY779559 | 13458 | B | A2 A24 B7 B13 | CA |
| AY779560 | 13458 | B | A2 A24 B7 B13 | CA |
| AY779561 | 13458 | B | A2 A24 B7 B13 | CA |
| AY779562 | 13458 | B | A2 A24 B7 B13 | CA |
| AY779563 | 13458 | B | A2 A24 B7 B13 | CA |
| AY779564 | 9869 | B | A2 A3 B57 B65 | CA |
| AY786790 | 9751 | B | A*03 A*31 B*08 B*15 Cw*04 Cw*07 | US |
| AY786791 | 9751 | B | A*03 A*31 B*08 B*15 Cw*04 Cw*07 | US |
| AY786792 | 9751 | B | A*03 A*31 B*08 B*15 Cw*04 Cw*07 | US |
| AY786793 | 9751 | B | A*03 A*31 B*08 B*15 Cw*04 Cw*07 | US |
| AY786794 | 9751 | B | A*03 A*31 B*08 B*15 Cw*04 Cw*07 | US |
| AY786795 | 9751 | B | A*03 A*31 B*08 B*15 Cw*04 Cw*07 | US |
| AY786796 | 9751 | B | A*03 A*31 B*08 B*15 Cw*04 Cw*07 | US |
| AY786797 | 9751 | B | A*03 A*31 B*08 B*15 Cw*04 Cw*07 | US |
| AY786798 | 9751 | B | A*03 A*31 B*08 B*15 Cw*04 Cw*07 | US |
| AY786799 | 9751 | B | A*03 A*31 B*08 B*15 Cw*04 Cw*07 | US |
| AY786800 | 9752 | B | A*24 A*31 B*47 B*15 Cw*04 Cw*07 | US |
| AY786801 | 9752 | B | A*24 A*31 B*47 B*15 Cw*04 Cw*07 | US |
| AY786802 | 9752 | B | A*24 A*31 B*47 B*15 Cw*04 Cw*07 | US |
| AY786803 | 9752 | B | A*24 A*31 B*47 B*15 Cw*04 Cw*07 | US |
| AY786804 | 9752 | B | A*24 A*31 B*47 B*15 Cw*04 Cw*07 | US |
| AY786805 | 9752 | B | A*24 A*31 B*47 B*15 Cw*04 Cw*07 | US |
| AY786806 | 9752 | B | A*24 A*31 B*47 B*15 Cw*04 Cw*07 | US |
| AY786807 | 9752 | B | A*24 A*31 B*47 B*15 Cw*04 Cw*07 | US |
| AY786808 | 9752 | B | A*24 A*31 B*47 B*15 Cw*04 Cw*07 | US |
| AY786809 | 9752 | B | A*24 A*31 B*47 B*15 Cw*04 Cw*07 | US |
| AY786810 | 9752 | B | A*24 A*31 B*47 B*15 Cw*04 Cw*07 | US |
| AY786811 | 9752 | B | A*24 A*31 B*47 B*15 Cw*04 Cw*07 | US |
| AY786812 | 9752 | B | A*24 A*31 B*47 B*15 Cw*04 Cw*07 | US |
| AY786813 | 9752 | B | A*24 A*31 B*47 B*15 Cw*04 Cw*07 | US |
| AY786814 | 9752 | B | A*24 A*31 B*47 B*15 Cw*04 Cw*07 | US |
| AY786815 | 9752 | B | A*24 A*31 B*47 B*15 Cw*04 Cw*07 | US |
| AY786816 | 9752 | B | A*24 A*31 B*47 B*15 Cw*04 Cw*07 | US |
| AY786817 | 9752 | B | A*24 A*31 B*47 B*15 Cw*04 Cw*07 | US |
| AY786818 | 9752 | B | A*24 A*31 B*47 B*15 Cw*04 Cw*07 | US |
| AY786819 | 9752 | B | A*24 A*31 B*47 B*15 Cw*04 Cw*07 | US |
| AY786820 | 9752 | B | A*24 A*31 B*47 B*15 Cw*04 Cw*07 | US |
| AY786821 | 9752 | B | A*24 A*31 B*47 B*15 Cw*04 Cw*07 | US |
| AY786822 | 9752 | B | A*24 A*31 B*47 B*15 Cw*04 Cw*07 | US |
| AY786823 | 9752 | B | A*24 A*31 B*47 B*15 Cw*04 Cw*07 | US |
| AY786824 | 9752 | B | A*24 A*31 B*47 B*15 Cw*04 Cw*07 | US |
| AY786825 | 9752 | B | A*24 A*31 B*47 B*15 Cw*04 Cw*07 | US |
| AY786826 | 9752 | B | A*24 A*31 B*47 B*15 Cw*04 Cw*07 | US |
| AY786827 | 9752 | B | A*24 A*31 B*47 B*15 Cw*04 Cw*07 | US |
| AY786828 | 9752 | B | A*24 A*31 B*47 B*15 Cw*04 Cw*07 | US |
| AY786829 | 9752 | B | A*24 A*31 B*47 B*15 Cw*04 Cw*07 | US |
| EF363125 | 13403 | B | A*02 A*03 B*5703 B*4402/4419 | US |
| EF363126 | 13403 | B | A*02 A*03 B*5703 B*4402/4419 | US |
| EU807832 | 28160 | B | A*02 A*30 B*5703 B*2703 | US |
| EU807833 | 28161 | B | A*330301 A*3402 B*440301 B*5703 | US |
| EU807834 | 28161 | B | A*330301 A*3402 B*440301 B*5703 | US |
| EU807835 | 28161 | B | A*330301 A*3402 B*440301 B*5703 | US |
| EU807836 | 28161 | B | A*330301 A*3402 B*440301 B*5703 | US |
| EU807837 | 28161 | B | A*330301 A*3402 B*440301 B*5703 | US |
| EU807838 | 28161 | B | A*330301 A*3402 B*440301 B*5703 | US |
| FJ495937 | 28466 | B | A*0101 A*2301 B*1402 B*5701 Cw*0701 Cw*0802 | US |
| FJ495939 | 28466 | B | A*0101 A*2301 B*1402 B*5701 Cw*0701 Cw*0802 | US |
| FJ495940 | 28466 | B | A*0101 A*2301 B*1402 B*5701 Cw*0701 Cw*0802 | US |
| FJ495941 | 28466 | B | A*0101 A*2301 B*1402 B*5701 Cw*0701 Cw*0802 | US |
| FJ495942 | 28466 | B | A*0101 A*2301 B*1402 B*5701 Cw*0701 Cw*0802 | US |
| FJ495943 | 28466 | B | A*0101 A*2301 B*1402 B*5701 Cw*0701 Cw*0802 | US |
| FJ495957 | 28466 | B | A*0101 A*2301 B*1402 B*5701 Cw*0701 Cw*0802 | US |
| FJ495958 | 28466 | B | A*0101 A*2301 B*1402 B*5701 Cw*0701 Cw*0802 | US |
| FJ495961 | 28466 | B | A*0101 A*2301 B*1402 B*5701 Cw*0701 Cw*0802 | US |
| FJ495962 | 28466 | B | A*0101 A*2301 B*1402 B*5701 Cw*0701 Cw*0802 | US |
| FJ495963 | 28466 | B | A*0101 A*2301 B*1402 B*5701 Cw*0701 Cw*0802 | US |
| FJ495973 | 28466 | B | A*0101 A*2301 B*1402 B*5701 Cw*0701 Cw*0802 | US |
| FJ495974 | 28466 | B | A*0101 A*2301 B*1402 B*5701 Cw*0701 Cw*0802 | US |
| FJ495975 | 28466 | B | A*0101 A*2301 B*1402 B*5701 Cw*0701 Cw*0802 | US |
| FJ495976 | 28466 | B | A*0101 A*2301 B*1402 B*5701 Cw*0701 Cw*0802 | US |
| FJ495977 | 28466 | B | A*0101 A*2301 B*1402 B*5701 Cw*0701 Cw*0802 | US |
| FJ495978 | 28466 | B | A*0101 A*2301 B*1402 B*5701 Cw*0701 Cw*0802 | US |
| FJ495979 | 28466 | B | A*0101 A*2301 B*1402 B*5701 Cw*0701 Cw*0802 | US |
| FJ495980 | 28466 | B | A*0101 A*2301 B*1402 B*5701 Cw*0701 Cw*0802 | US |
| FJ495981 | 28466 | B | A*0101 A*2301 B*1402 B*5701 Cw*0701 Cw*0802 | US |
| FJ495991 | 28466 | B | A*0101 A*2301 B*1402 B*5701 Cw*0701 Cw*0802 | US |
| FJ495992 | 28466 | B | A*0101 A*2301 B*1402 B*5701 Cw*0701 Cw*0802 | US |
| FJ495993 | 28466 | B | A*0101 A*2301 B*1402 B*5701 Cw*0701 Cw*0802 | US |
| FJ495994 | 28466 | B | A*0101 A*2301 B*1402 B*5701 Cw*0701 Cw*0802 | US |
| FJ495995 | 28466 | B | A*0101 A*2301 B*1402 B*5701 Cw*0701 Cw*0802 | US |
| FJ495996 | 28466 | B | A*0101 A*2301 B*1402 B*5701 Cw*0701 Cw*0802 | US |
| FJ495997 | 28466 | B | A*0101 A*2301 B*1402 B*5701 Cw*0701 Cw*0802 | US |
| FJ495998 | 28466 | B | A*0101 A*2301 B*1402 B*5701 Cw*0701 Cw*0802 | US |
| FJ495999 | 28466 | B | A*0101 A*2301 B*1402 B*5701 Cw*0701 Cw*0802 | US |
| FJ496000 | 28467 | B | A*0205 A*0205 B*5301 B*5701 Cw*0401 Cw*1801 | US |
| FJ496001 | 28467 | B | A*0205 A*0205 B*5301 B*5701 Cw*0401 Cw*1801 | US |
| FJ496002 | 28467 | B | A*0205 A*0205 B*5301 B*5701 Cw*0401 Cw*1801 | US |
| FJ496003 | 28467 | B | A*0205 A*0205 B*5301 B*5701 Cw*0401 Cw*1801 | US |
| FJ496004 | 28467 | B | A*0205 A*0205 B*5301 B*5701 Cw*0401 Cw*1801 | US |
| FJ496005 | 28467 | B | A*0205 A*0205 B*5301 B*5701 Cw*0401 Cw*1801 | US |
| FJ496006 | 28467 | B | A*0205 A*0205 B*5301 B*5701 Cw*0401 Cw*1801 | US |
| FJ496007 | 28467 | B | A*0205 A*0205 B*5301 B*5701 Cw*0401 Cw*1801 | US |
| FJ496024 | 28467 | B | A*0205 A*0205 B*5301 B*5701 Cw*0401 Cw*1801 | US |
| FJ496025 | 28467 | B | A*0205 A*0205 B*5301 B*5701 Cw*0401 Cw*1801 | US |
| FJ496026 | 28467 | B | A*0205 A*0205 B*5301 B*5701 Cw*0401 Cw*1801 | US |
| FJ496027 | 28467 | B | A*0205 A*0205 B*5301 B*5701 Cw*0401 Cw*1801 | US |
| FJ496033 | 28467 | B | A*0205 A*0205 B*5301 B*5701 Cw*0401 Cw*1801 | US |
| FJ496034 | 28467 | B | A*0205 A*0205 B*5301 B*5701 Cw*0401 Cw*1801 | US |
| FJ496035 | 28467 | B | A*0205 A*0205 B*5301 B*5701 Cw*0401 Cw*1801 | US |
| FJ496036 | 28467 | B | A*0205 A*0205 B*5301 B*5701 Cw*0401 Cw*1801 | US |
| FJ496037 | 28467 | B | A*0205 A*0205 B*5301 B*5701 Cw*0401 Cw*1801 | US |
| FJ496038 | 28467 | B | A*0205 A*0205 B*5301 B*5701 Cw*0401 Cw*1801 | US |
| FJ496039 | 28467 | B | A*0205 A*0205 B*5301 B*5701 Cw*0401 Cw*1801 | US |
| FJ496040 | 28467 | B | A*0205 A*0205 B*5301 B*5701 Cw*0401 Cw*1801 | US |
| FJ496041 | 28467 | B | A*0205 A*0205 B*5301 B*5701 Cw*0401 Cw*1801 | US |
| FJ496058 | 28467 | B | A*0205 A*0205 B*5301 B*5701 Cw*0401 Cw*1801 | US |
| FJ496059 | 28467 | B | A*0205 A*0205 B*5301 B*5701 Cw*0401 Cw*1801 | US |
| FJ496068 | 28467 | B | A*0205 A*0205 B*5301 B*5701 Cw*0401 Cw*1801 | US |
| FJ496069 | 28467 | B | A*0205 A*0205 B*5301 B*5701 Cw*0401 Cw*1801 | US |
| FJ496070 | 28467 | B | A*0205 A*0205 B*5301 B*5701 Cw*0401 Cw*1801 | US |
| FJ496071 | 28467 | B | A*0205 A*0205 B*5301 B*5701 Cw*0401 Cw*1801 | US |
| FJ496123 | 28467 | B | A*0205 A*0205 B*5301 B*5701 Cw*0401 Cw*1801 | US |
| FJ496128 | 28467 | B | A*0205 A*0205 B*5301 B*5701 Cw*0401 Cw*1801 | US |
| FJ496130 | 28467 | B | A*0205 A*0205 B*5301 B*5701 Cw*0401 Cw*1801 | US |
| FJ496131 | 28467 | B | A*0205 A*0205 B*5301 B*5701 Cw*0401 Cw*1801 | US |
| FJ496132 | 28467 | B | A*0205 A*0205 B*5301 B*5701 Cw*0401 Cw*1801 | US |
| FJ496133 | 28467 | B | A*0205 A*0205 B*5301 B*5701 Cw*0401 Cw*1801 | US |
| FJ496134 | 28467 | B | A*0205 A*0205 B*5301 B*5701 Cw*0401 Cw*1801 | US |
| FJ496135 | 28467 | B | A*0205 A*0205 B*5301 B*5701 Cw*0401 Cw*1801 | US |
| FJ496136 | 28467 | B | A*0205 A*0205 B*5301 B*5701 Cw*0401 Cw*1801 | US |
| FJ919955 | 28467 | B | A*0205 A*0205 B*5301 B*5701 Cw*0401 Cw*1801 | US |
| FJ919956 | 28467 | B | A*0205 A*0205 B*5301 B*5701 Cw*0401 Cw*1801 | US |
| FJ919957 | 28467 | B | A*0205 A*0205 B*5301 B*5701 Cw*0401 Cw*1801 | US |
| FJ919958 | 28467 | B | A*0205 A*0205 B*5301 B*5701 Cw*0401 Cw*1801 | US |
| FJ919959 | 28467 | B | A*0205 A*0205 B*5301 B*5701 Cw*0401 Cw*1801 | US |
| FJ919960 | 28467 | B | A*0205 A*0205 B*5301 B*5701 Cw*0401 Cw*1801 | US |
| FJ919961 | 28467 | B | A*0205 A*0205 B*5301 B*5701 Cw*0401 Cw*1801 | US |
| FJ919962 | 28467 | B | A*0205 A*0205 B*5301 B*5701 Cw*0401 Cw*1801 | US |
| JF320363 | 39593 | B | B*5701 CW*0702 A*0301 CW*0602 A*0101 B*0702 | US |
| JF320364 | 39593 | B | B*5701 CW*0702 A*0301 CW*0602 A*0101 B*0702 | US |
| JF320365 | 39593 | B | B*5701 CW*0702 A*0301 CW*0602 A*0101 B*0702 | US |
| JF320366 | 39593 | B | B*5701 CW*0702 A*0301 CW*0602 A*0101 B*0702 | US |
| JF320369 | 39593 | B | B*5701 CW*0702 A*0301 CW*0602 A*0101 B*0702 | US |
| JF320373 | 39593 | B | B*5701 CW*0702 A*0301 CW*0602 A*0101 B*0702 | US |
| JF320374 | 39593 | B | B*5701 CW*0702 A*0301 CW*0602 A*0101 B*0702 | US |
| JF320514 | 39593 | B | B*5701 CW*0702 A*0301 CW*0602 A*0101 B*0702 | US |
| JF320515 | 39593 | B | B*5701 CW*0702 A*0301 CW*0602 A*0101 B*0702 | US |
| JF320516 | 39593 | B | B*5701 CW*0702 A*0301 CW*0602 A*0101 B*0702 | US |
| JF320518 | 39593 | B | B*5701 CW*0702 A*0301 CW*0602 A*0101 B*0702 | US |
| JF320519 | 39593 | B | B*5701 CW*0702 A*0301 CW*0602 A*0101 B*0702 | US |
| JF320559 | 39645 | B | B*5701 CW*0802 A*2910 CW*0602 A*0101 B*1402 | US |
| JF320561 | 39645 | B | B*5701 CW*0802 A*2910 CW*0602 A*0101 B*1402 | US |
| JF320562 | 39645 | B | B*5701 CW*0802 A*2910 CW*0602 A*0101 B*1402 | US |
| JF320563 | 39645 | B | B*5701 CW*0802 A*2910 CW*0602 A*0101 B*1402 | US |
| AY786830 | 9753 | B | A*30 B*18 B*40 Cw*02 Cw*05 | US |
| AY786831 | 9753 | B | A*30 B*18 B*40 Cw*02 Cw*05 | US |
| AY786832 | 9753 | B | A*30 B*18 B*40 Cw*02 Cw*05 | US |
| AY786833 | 9753 | B | A*30 B*18 B*40 Cw*02 Cw*05 | US |
| AY786834 | 9753 | B | A*30 B*18 B*40 Cw*02 Cw*05 | US |
| AY786835 | 9753 | B | A*30 B*18 B*40 Cw*02 Cw*05 | US |
| AY786836 | 9753 | B | A*30 B*18 B*40 Cw*02 Cw*05 | US |
| AY786837 | 9753 | B | A*30 B*18 B*40 Cw*02 Cw*05 | US |
| AY786838 | 9753 | B | A*30 B*18 B*40 Cw*02 Cw*05 | US |
| AY786839 | 9753 | B | A*30 B*18 B*40 Cw*02 Cw*05 | US |
| AY786840 | 9754 | B | A*02 A*30 B*18 B*13 Cw*01 Cw*05 | US |
| AY786841 | 9754 | B | A*02 A*30 B*18 B*13 Cw*01 Cw*05 | US |
| AY786842 | 9754 | B | A*02 A*30 B*18 B*13 Cw*01 Cw*05 | US |
| AY786843 | 9754 | B | A*02 A*30 B*18 B*13 Cw*01 Cw*05 | US |
| AY786844 | 9754 | B | A*02 A*30 B*18 B*13 Cw*01 Cw*05 | US |
| AY786845 | 9754 | B | A*02 A*30 B*18 B*13 Cw*01 Cw*05 | US |
| AY786846 | 9754 | B | A*02 A*30 B*18 B*13 Cw*01 Cw*05 | US |
| AY786847 | 9754 | B | A*02 A*30 B*18 B*13 Cw*01 Cw*05 | US |
| AY786848 | 9754 | B | A*02 A*30 B*18 B*13 Cw*01 Cw*05 | US |
| AY786849 | 9754 | B | A*02 A*30 B*18 B*13 Cw*01 Cw*05 | US |
| AY786850 | 9754 | B | A*02 A*30 B*18 B*13 Cw*01 Cw*05 | US |
| AY786851 | 9754 | B | A*02 A*30 B*18 B*13 Cw*01 Cw*05 | US |
| AY786852 | 9754 | B | A*02 A*30 B*18 B*13 Cw*01 Cw*05 | US |
| AY786853 | 9754 | B | A*02 A*30 B*18 B*13 Cw*01 Cw*05 | US |
| AY786854 | 9754 | B | A*02 A*30 B*18 B*13 Cw*01 Cw*05 | US |
| AY786855 | 9754 | B | A*02 A*30 B*18 B*13 Cw*01 Cw*05 | US |
| AY786856 | 9754 | B | A*02 A*30 B*18 B*13 Cw*01 Cw*05 | US |
| AY786857 | 9754 | B | A*02 A*30 B*18 B*13 Cw*01 Cw*05 | US |
| AY786858 | 9754 | B | A*02 A*30 B*18 B*13 Cw*01 Cw*05 | US |
| AY786859 | 9754 | B | A*02 A*30 B*18 B*13 Cw*01 Cw*05 | US |
| AY786860 | 9754 | B | A*02 A*30 B*18 B*13 Cw*01 Cw*05 | US |
| AY786861 | 9754 | B | A*02 A*30 B*18 B*13 Cw*01 Cw*05 | US |
| AY786862 | 9754 | B | A*02 A*30 B*18 B*13 Cw*01 Cw*05 | US |
| AY786863 | 9754 | B | A*02 A*30 B*18 B*13 Cw*01 Cw*05 | US |
| AY786864 | 9754 | B | A*02 A*30 B*18 B*13 Cw*01 Cw*05 | US |
| AY786865 | 9754 | B | A*02 A*30 B*18 B*13 Cw*01 Cw*05 | US |
| AY786866 | 9754 | B | A*02 A*30 B*18 B*13 Cw*01 Cw*05 | US |
| AY786867 | 9754 | B | A*02 A*30 B*18 B*13 Cw*01 Cw*05 | US |
| AY786868 | 9754 | B | A*02 A*30 B*18 B*13 Cw*01 Cw*05 | US |
| AY786869 | 9754 | B | A*02 A*30 B*18 B*13 Cw*01 Cw*05 | US |
| AY786870 | 9755 | B | A*24 A*30 B*39 B*47 Cw*12 Cw*17 | US |
| AY786871 | 9755 | B | A*24 A*30 B*39 B*47 Cw*12 Cw*17 | US |
| AY786872 | 9755 | B | A*24 A*30 B*39 B*47 Cw*12 Cw*17 | US |
| AY786873 | 9755 | B | A*24 A*30 B*39 B*47 Cw*12 Cw*17 | US |
| AY786874 | 9755 | B | A*24 A*30 B*39 B*47 Cw*12 Cw*17 | US |
| AY786875 | 9755 | B | A*24 A*30 B*39 B*47 Cw*12 Cw*17 | US |
| AY786876 | 9755 | B | A*24 A*30 B*39 B*47 Cw*12 Cw*17 | US |
| AY786877 | 9755 | B | A*24 A*30 B*39 B*47 Cw*12 Cw*17 | US |
| AY786878 | 9755 | B | A*24 A*30 B*39 B*47 Cw*12 Cw*17 | US |
| AY786879 | 9755 | B | A*24 A*30 B*39 B*47 Cw*12 Cw*17 | US |
| AY786880 | 15306 | B | A*24 A*23 B*39 B*07 Cw*12 Cw*17 | US |
| AY786881 | 15306 | B | A*24 A*23 B*39 B*07 Cw*12 Cw*17 | US |
| AY786882 | 15306 | B | A*24 A*23 B*39 B*07 Cw*12 Cw*17 | US |
| AY786883 | 15306 | B | A*24 A*23 B*39 B*07 Cw*12 Cw*17 | US |
| AY786884 | 15306 | B | A*24 A*23 B*39 B*07 Cw*12 Cw*17 | US |
| AY786885 | 15306 | B | A*24 A*23 B*39 B*07 Cw*12 Cw*17 | US |
| AY786886 | 15306 | B | A*24 A*23 B*39 B*07 Cw*12 Cw*17 | US |
| AY786887 | 15306 | B | A*24 A*23 B*39 B*07 Cw*12 Cw*17 | US |
| AY786888 | 15306 | B | A*24 A*23 B*39 B*07 Cw*12 Cw*17 | US |
| AY786889 | 15306 | B | A*24 A*23 B*39 B*07 Cw*12 Cw*17 | US |
| AY786890 | 15306 | B | A*24 A*23 B*39 B*07 Cw*12 Cw*17 | US |
| AY786891 | 15306 | B | A*24 A*23 B*39 B*07 Cw*12 Cw*17 | US |
| AY786892 | 15306 | B | A*24 A*23 B*39 B*07 Cw*12 Cw*17 | US |
| AY786893 | 15306 | B | A*24 A*23 B*39 B*07 Cw*12 Cw*17 | US |
| AY786894 | 15306 | B | A*24 A*23 B*39 B*07 Cw*12 Cw*17 | US |
| AY786895 | 15306 | B | A*24 A*23 B*39 B*07 Cw*12 Cw*17 | US |
| AY786896 | 15306 | B | A*24 A*23 B*39 B*07 Cw*12 Cw*17 | US |
| AY786897 | 15306 | B | A*24 A*23 B*39 B*07 Cw*12 Cw*17 | US |
| AY786898 | 15306 | B | A*24 A*23 B*39 B*07 Cw*12 Cw*17 | US |
| AY786899 | 15306 | B | A*24 A*23 B*39 B*07 Cw*12 Cw*17 | US |
| AY786900 | 15306 | B | A*24 A*23 B*39 B*07 Cw*12 Cw*17 | US |
| AY786901 | 15306 | B | A*24 A*23 B*39 B*07 Cw*12 Cw*17 | US |
| AY786902 | 15306 | B | A*24 A*23 B*39 B*07 Cw*12 Cw*17 | US |
| AY786903 | 15306 | B | A*24 A*23 B*39 B*07 Cw*12 Cw*17 | US |
| AY786904 | 15306 | B | A*24 A*23 B*39 B*07 Cw*12 Cw*17 | US |
| DQ487190 | 21249 | B | A1 A19 B*3501 B44 Cw7 Cw16 | US |
| DQ487191 | 21249 | B | A1 A19 B*3501 B44 Cw7 Cw16 | US |
| HM208363 | 36113 | B | B*27, B*35, A*24, A*30 | US |
| HM586191 | 36106 | B | A*02 A*03 B*15 B*35 Cw*09 Cw*04 | GB |
| HM586193 | 36106 | B | A*02 A*03 B*15 B*35 Cw*09 Cw*04 | GB |
| HM586194 | 36106 | B | A*02 A*03 B*15 B*35 Cw*09 Cw*04 | GB |
| HM586196 | 36106 | B | A*02 A*03 B*15 B*35 Cw*09 Cw*04 | GB |
| JF320028 | 39682 | B | B*4064 CW*0401 A*2402 CW*0304 A*0201 B*3520 | PE |
| JF320029 | 39682 | B | B*4064 CW*0401 A*2402 CW*0304 A*0201 B*3520 | PE |
| JF320031 | 39682 | B | B*4064 CW*0401 A*2402 CW*0304 A*0201 B*3520 | PE |
| JF320032 | 39682 | B | B*4064 CW*0401 A*2402 CW*0304 A*0201 B*3520 | PE |
| JF320044 | 39591 | B | B*3501 CW*0401 A*2402 CW*0303 A*0206 B*2705 | US |
| JF320047 | 39591 | B | B*3501 CW*0401 A*2402 CW*0303 A*0206 B*2705 | US |
| JF320048 | 39733 | B | B*4901 CW*0701 A*2402 CW*0102 A*2301 B*3543 | US |
| JF320049 | 39591 | B | B*3501 CW*0401 A*2402 CW*0303 A*0206 B*2705 | US |
| JF320051 | 39591 | B | B*3501 CW*0401 A*2402 CW*0303 A*0206 B*2705 | US |
| JF320053 | 39591 | B | B*3501 CW*0401 A*2402 CW*0303 A*0206 B*2705 | US |
| JF320055 | 39591 | B | B*3501 CW*0401 A*2402 CW*0303 A*0206 B*2705 | US |
| JF320056 | 39733 | B | B*4901 CW*0701 A*2402 CW*0102 A*2301 B*3543 | US |
| JF320058 | 39591 | B | B*3501 CW*0401 A*2402 CW*0303 A*0206 B*2705 | US |
| JF320059 | 39731 | B | B*3501 CW*0401 A*2402 CW*0202 A*0201 B*2705 | US |
| JF320060 | 39591 | B | B*3501 CW*0401 A*2402 CW*0303 A*0206 B*2705 | US |
| JF320062 | 39591 | B | B*3501 CW*0401 A*2402 CW*0303 A*0206 B*2705 | US |
| JF320064 | 39591 | B | B*3501 CW*0401 A*2402 CW*0303 A*0206 B*2705 | US |
| JF320065 | 39733 | B | B*4901 CW*0701 A*2402 CW*0102 A*2301 B*3543 | US |
| JF320068 | 39733 | B | B*4901 CW*0701 A*2402 CW*0102 A*2301 B*3543 | US |
| JF320071 | 39733 | B | B*4901 CW*0701 A*2402 CW*0102 A*2301 B*3543 | US |
| JF320145 | 39631 | B | B*5305 A*6802 CW*0401 A*0301 B*3527 | US |
| JF320147 | 39631 | B | B*5305 A*6802 CW*0401 A*0301 B*3527 | US |
| JF320152 | 39631 | B | B*5305 A*6802 CW*0401 A*0301 B*3527 | US |
| JF320153 | 39631 | B | B*5305 A*6802 CW*0401 A*0301 B*3527 | US |
| JF320154 | 39631 | B | B*5305 A*6802 CW*0401 A*0301 B*3527 | US |
| JF320179 | 39693 | B | B*3501 CW*0701 A*1101 CW*0401 A*0101 B*0801 | US |
| JF320181 | 39693 | B | B*3501 CW*0701 A*1101 CW*0401 A*0101 B*0801 | US |
| JF320182 | 39693 | B | B*3501 CW*0701 A*1101 CW*0401 A*0101 B*0801 | US |
| JF320183 | 39708 | B | B*5701 CW*0602 A*9205 CW*0304 A*0101 B*3501 | PE |
| JF320185 | 39730 | B | B*3501 CW*0602 CW*0202 A*6802 B*1801 | US |
| JF320186 | 39620 | B | B*3543 CW*0401 A*3101 CW*0102 A*0211 B*3520 | PE |
| JF320187 | 39708 | B | B*5701 CW*0602 A*9205 CW*0304 A*0101 B*3501 | PE |
| JF320188 | 39730 | B | B*3501 CW*0602 CW*0202 A*6802 B*1801 | US |
| JF320190 | 39730 | B | B*3501 CW*0602 CW*0202 A*6802 B*1801 | US |
| JF320192 | 39730 | B | B*3501 CW*0602 CW*0202 A*6802 B*1801 | US |
| JF320193 | 39620 | B | B*3543 CW*0401 A*3101 CW*0102 A*0211 B*3520 | PE |
| JF320194 | 39730 | B | B*3501 CW*0602 CW*0202 A*6802 B*1801 | US |
| JF320197 | 39615 | B | B*3517 CW*0702 A*2601 CW*0401 A*0201 B*0702 | US |
| JF320200 | 39615 | B | B*3517 CW*0702 A*2601 CW*0401 A*0201 B*0702 | US |
| JF320201 | 39620 | B | B*3543 CW*0401 A*3101 CW*0102 A*0211 B*3520 | PE |
| JF320202 | 39615 | B | B*3517 CW*0702 A*2601 CW*0401 A*0201 B*0702 | US |
| JF320205 | 39615 | B | B*3517 CW*0702 A*2601 CW*0401 A*0201 B*0702 | US |
| JF320207 | 39615 | B | B*3517 CW*0702 A*2601 CW*0401 A*0201 B*0702 | US |
| JF320209 | 39708 | B | B*5701 CW*0602 A*9205 CW*0304 A*0101 B*3501 | PE |
| JF320212 | 39708 | B | B*5701 CW*0602 A*9205 CW*0304 A*0101 B*3501 | PE |
| JF320215 | 39620 | B | B*3543 CW*0401 A*3101 CW*0102 A*0211 B*3520 | PE |
| JF320307 | 39669 | B | B*3501 A*2402 CW*0303 A*0201 B*1501 | US |
| JF320309 | 39669 | B | B*3501 A*2402 CW*0303 A*0201 B*1501 | US |
| JF320311 | 39669 | B | B*3501 A*2402 CW*0303 A*0201 B*1501 | US |
| JF320381 | 39719 | B | B*4901 CW*0701 A*6801 CW*0401 A*0205 B*3501 | US |
| JF320384 | 39719 | B | B*4901 CW*0701 A*6801 CW*0401 A*0205 B*3501 | US |
| JF320409 | 39664 | B | B*5101 CW*1502 A*3101 CW*0401 A*1101 B*3503 | US |
| JF320460 | 39608 | B | B*5101 CW*1502 A*2402 C*0404 A*2301 B*3502 | US |
| JF320461 | 39608 | B | B*5101 CW*1502 A*2402 C*0404 A*2301 B*3502 | US |
| JF320462 | 39608 | B | B*5101 CW*1502 A*2402 C*0404 A*2301 B*3502 | US |
| JF320463 | 39608 | B | B*5101 CW*1502 A*2402 C*0404 A*2301 B*3502 | US |
| JF320464 | 39608 | B | B*5101 CW*1502 A*2402 C*0404 A*2301 B*3502 | US |
| JF320465 | 39608 | B | B*5101 CW*1502 A*2402 C*0404 A*2301 B*3502 | US |
| JF320466 | 39608 | B | B*5101 CW*1502 A*2402 C*0404 A*2301 B*3502 | US |
| JF320467 | 39608 | B | B*5101 CW*1502 A*2402 C*0404 A*2301 B*3502 | US |
| JF320468 | 39608 | B | B*5101 CW*1502 A*2402 C*0404 A*2301 B*3502 | US |
| JF320469 | 39608 | B | B*5101 CW*1502 A*2402 C*0404 A*2301 B*3502 | US |
| JF320470 | 39608 | B | B*5101 CW*1502 A*2402 C*0404 A*2301 B*3502 | US |
| JF320569 | 39664 | B | B*5101 CW*1502 A*3101 CW*0401 A*1101 B*3503 | US |
| JF320571 | 39664 | B | B*5101 CW*1502 A*3101 CW*0401 A*1101 B*3503 | US |
| JF320572 | 39664 | B | B*5101 CW*1502 A*3101 CW*0401 A*1101 B*3503 | US |
| JF320573 | 39664 | B | B*5101 CW*1502 A*3101 CW*0401 A*1101 B*3503 | US |
| JF320574 | 39664 | B | B*5101 CW*1502 A*3101 CW*0401 A*1101 B*3503 | US |
| JF320575 | 39664 | B | B*5101 CW*1502 A*3101 CW*0401 A*1101 B*3503 | US |
| JF320576 | 39664 | B | B*5101 CW*1502 A*3101 CW*0401 A*1101 B*3503 | US |
| JF320577 | 39664 | B | B*5101 CW*1502 A*3101 CW*0401 A*1101 B*3503 | US |
| JF320582 | 39664 | B | B*5101 CW*1502 A*3101 CW*0401 A*1101 B*3503 | US |
| JF320591 | 39664 | B | B*5101 CW*1502 A*3101 CW*0401 A*1101 B*3503 | US |
| JF320615 | 39734 | B | B*4501 CW*0602 A*2902 CW*0401 A*1101 B*3501 | US |
| JF320617 | 39734 | B | B*4501 CW*0602 A*2902 CW*0401 A*1101 B*3501 | US |
| JF320620 | 39734 | B | B*4501 CW*0602 A*2902 CW*0401 A*1101 B*3501 | US |
| JF320621 | 39734 | B | B*4501 CW*0602 A*2902 CW*0401 A*1101 B*3501 | US |
| JF320623 | 39734 | B | B*4501 CW*0602 A*2902 CW*0401 A*1101 B*3501 | US |
| JF320624 | 39734 | B | B*4501 CW*0602 A*2902 CW*0401 A*1101 B*3501 | US |
| JF320625 | 39734 | B | B*4501 CW*0602 A*2902 CW*0401 A*1101 B*3501 | US |
| JF320626 | 39734 | B | B*4501 CW*0602 A*2902 CW*0401 A*1101 B*3501 | US |
| JF320627 | 39734 | B | B*4501 CW*0602 A*2902 CW*0401 A*1101 B*3501 | US |
