# Supplementary material for: Detecting consistent patterns of directional adaptation using differential selection codon models
Source: BMC Evol Biol. 2017 Jun 23;17:147. doi: 10.1186/s12862-017-0979-y (PMC5481935; doi:10.1186/s12862-017-0979-y)
Supplement: Supplementary file 2 — Table S2. Newick format of tree T1, T2 and T3. (DOCX 18 kb) [file 12862_2017_979_MOESM2_ESM.docx]

Table S 2. Newick format of tree T1, T2 and T3.

| **T1** |
| --- |

((((((FJ496035:2,(((((FJ496040:2,(FJ496041:2,FJ496033:2):2):2,FJ496036:2):2,FJ496038:2):2,FJ496034:2):2,FJ496037:2):2):2,((((FJ919955:2,(FJ919958:2,FJ919959:2):2):2,FJ919960:2):2,FJ919956:2):2,((FJ919961:2,FJ919962:2):2,FJ919957:2):2):2):2,(((FJ496025:2,FJ496027:2):2,FJ496039:2):2,(FJ496134:2,(FJ496058:2,(FJ496069:2,(FJ496128:2,(FJ496123:2,(FJ496071:2,(FJ496135:2,(FJ496131:2,(FJ496059:2,(FJ496132:2,(FJ496005:2,(FJ496003:2,(FJ496004:2,(FJ496133:2,(FJ496000:2,(((((FJ496068:2,FJ496136:2):2,(FJ496002:2,FJ496006:2):2):2,(FJ496007:2,FJ496001:2):2):2,(FJ496024:2,FJ496026:2):2):2,(FJ496070:2,FJ496130:2):2):2):2):2):2):2):2):2):2):2):2):2):2):2):2):2):2):2):2,(AY331290:1,AY331289:1):1):0,((((((((((((AY786889:1,AY786883:1):1,(((AY786885:1,AY786880:1):1,AY786888:1):1,((((((((AY786895:1,(AY786893:1,AY786896:1):1):1,AY786894:1):1,AY786891:1):1,AY786899:1):1,AY786890:1):1,AY786898:1):1,AY786897:1):1,AY786892:1):1):1):1,AY786904:1):1,(((AY786881:1,(AY786886:1,AY786887:1):1):1,AY786884:1):1,(AY786900:1,AY786901:1):1):1):1,(((((AY779562:1,AY779557:1):1,AY779558:1):1,AY779563:1):1,(AY779560:1,(AY779561:1,AY779559:1):1):1):1,(EF363126:2,EF363125:2):2):0):0,((((JF320200:3,JF320202:3):3,JF320197:3):3,JF320207:3):3,JF320205:3):3):0,((((((AY423382:1,AY423384:1):1,AY423386:1):1,AY423385:1):1,AY423381:1):1,((((((((((AY786831:1,AY786837:1):1,AY786839:1):1,AY786832:1):1,AY786830:1):1,AY786835:1):1,AY786833:1):1,AY786834:1):1,AY786838:1):1,AY786836:1):1,((((((((((((AY786843:1,(AY786842:1,AY786847:1):1):1,AY786841:1):1,AY786840:1):1,(AY786844:1,AY786848:1):1):1,AY786845:1):1,AY786849:1):1,(AY786852:1,AY786854:1):1):1,AY786846:1):1,AY786851:1):1,((AY786869:1,(AY786856:1,AY786853:1):1):1,AY786866:1):1):1,(((AY786868:1,AY786867:1):1,AY786863:1):1,(AY786861:1,AY786864:1):1):1):1,AY786860:1):1):0):0,(((AY331282:1,AY331283:1):1,((DQ487191:3,DQ487190:3):3,((((((EU807836:2,EU807838:2):2,EU807835:2):2,EU807834:2):2,EU807833:2):2,EU807837:2):2,EU807832:2):0):0):0,((JF320307:3,JF320309:3):3,JF320311:3):3):0):0):0,(((((JF320627:3,(JF320626:3,(JF320615:3,(JF320617:3,(JF320620:3,(JF320623:3,(JF320624:3,JF320625:3):3):3):3):3):3):3):3,JF320621:3):3,(JF320059:3,(((HM586193:3,HM586191:3):3,HM586194:3):3,HM586196:3):3):0):0,((JF320062:3,((((JF320051:3,JF320064:3):3,JF320047:3):3,JF320044:3):3,JF320049:3):3):3,((JF320058:3,(JF320060:3,JF320053:3):3):3,JF320055:3):3):3):0,((((JF320179:3,JF320182:3):3,JF320181:3):3,((JF320187:2,(JF320209:2,JF320212:2):2):2,JF320183:2):2):0,(((JF320193:3,JF320186:3):3,JF320215:3):3,JF320201:3):3):0):0):0,(((((JF320563:2,JF320562:2):2,JF320559:2):2,JF320561:2):2,(((JF320031:3,JF320028:3):3,(JF320029:3,JF320032:3):3):3,(((FJ495963:2,FJ495961:2):2,FJ495962:2):2,(((((((((((((FJ495937:2,(FJ495978:2,(FJ495994:2,(FJ495979:2,FJ495975:2):2):2):2):2,FJ495974:2):2,FJ495981:2):2,FJ495941:2):2,FJ495939:2):2,FJ495943:2):2,FJ495973:2):2,FJ495940:2):2,FJ495942:2):2,(((((FJ495977:2,FJ495992:2):2,FJ495995:2):2,FJ495997:2):2,FJ495998:2):2,FJ495996:2):2):2,((FJ495991:2,(FJ495958:2,FJ495993:2):2):2,FJ495976:2):2):2,FJ495980:2):2,(FJ495999:2,FJ495957:2):2):2):2):0):0,((AY331293:1,HM208363:3):0,((((((JF320463:3,JF320466:3):3,(((JF320461:3,JF320468:3):3,(((JF320467:3,JF320460:3):3,JF320469:3):3,JF320470:3):3):3,JF320464:3):3):3,(JF320465:3,JF320462:3):3):3,(AY331284:1,AY332236:1):1):0,(JF320071:3,(((JF320048:3,JF320056:3):3,JF320068:3):3,JF320065:3):3):3):0,(((((JF320518:2,(JF320519:2,(JF320364:2,(JF320366:2,(JF320365:2,JF320363:2):2):2):2):2):2,JF320373:2):2,JF320369:2):2,(((JF320516:2,JF320514:2):2,JF320515:2):2,JF320374:2):2):2,(JF320384:3,JF320381:3):3):0):0):0):0):0,(AY331286:1,AY331285:1):1):0,((((AY779553:1,AY779554:1):1,AY779555:1):1,((AY779551:1,(AY779564:1,AY779550:1):1):1,AY779552:1):1):0,((JF320154:3,(JF320147:3,(JF320145:3,JF320153:3):3):3):3,JF320152:3):3):0):0,(((JF320185:3,(((JF320188:3,JF320190:3):3,JF320194:3):3,JF320192:3):3):3,AY331287:1):0,((((((JF320569:3,JF320582:3):3,JF320409:3):3,JF320591:3):3,(JF320572:3,JF320571:3):3):3,(((JF320576:3,JF320573:3):3,JF320574:3):3,JF320575:3):3):3,JF320577:3):3):0):0):0,((((((((((((AY786800:1,AY786802:1):1,AY786803:1):1,AY786804:1):1,AY786808:1):1,AY786801:1):1,AY786805:1):1,(AY786809:1,AY786807:1):1):1,AY786806:1):1,(((((((((AY786813:1,AY786817:1):1,AY786810:1):1,AY786811:1):1,AY786818:1):1,AY786819:1):1,AY786814:1):1,AY786815:1):1,AY786812:1):1,AY786816:1):1):1,((((((AY786823:1,((AY786829:1,AY786824:1):1,AY786821:1):1):1,AY786828:1):1,(AY786825:1,AY786820:1):1):1,AY786826:1):1,AY786827:1):1,AY786822:1):1):1,((AY786792:1,((((AY786793:1,AY786795:1):1,AY786796:1):1,AY786798:1):1,AY786799:1):1):1,(((AY786794:1,AY786790:1):1,AY786791:1):1,AY786797:1):1):1):0,(AY331296:1,AY331297:1):1):0);

| **T2** |
| --- |

(AY331282:1.0,AY331283:1.0,(((AY331284:1.0,AY332236:1.0):1.0,((AY331287:1.0,(HM586191:3.0,HM586193:3.0,HM586194:3.0,HM586196:3.0):3.0):0.0,(JF320185:3.0,JF320188:3.0,JF320190:3.0,JF320192:3.0,JF320194:3.0):3.0):0.0):0.0,(AY331285:1.0,AY331286:1.0):1.0,((AY331289:1.0,AY331290:1.0):1.0,(((((AY786880:1.0,(AY786882:1.0,AY786885:1.0):1.0,AY786888:1.0):1.0,AY786902:1.0):1.0,(((AY786881:1.0,(AY786886:1.0,AY786887:1.0):1.0):1.0,AY786884:1.0):1.0,(AY786890:1.0,AY786891:1.0,(AY786892:1.0,AY786893:1.0,AY786895:1.0,AY786896:1.0,AY786899:1.0):1.0,AY786894:1.0,AY786897:1.0,AY786898:1.0):1.0):1.0,(AY786883:1.0,AY786889:1.0):1.0):1.0,(AY786900:1.0,AY786901:1.0):1.0,(AY786903:1.0,AY786904:1.0):1.0):1.0,(AY786879:1.0,AY786871:1.0,AY786870:1.0,AY786875:1.0,AY786872:1.0,AY786877:1.0,AY786878:1.0,AY786873:1.0,AY786874:1.0,AY786876:1.0):1.0):0.0):0.0,(AY331293:1.0,(AY331296:1.0,AY331297:1.0):1.0):0.0,(AY423381:1.0,AY423382:1.0,(AY423384:1.0,(AY423385:1.0,AY423386:1.0):1.0):1.0):1.0,(((((AY779550:1.0,AY779551:1.0):1.0,AY779564:1.0):1.0,AY779552:1.0):1.0,((AY779553:1.0,AY779555:1.0):1.0,AY779554:1.0):1.0):0.0,((((((AY779557:1.0,AY779562:1.0):1.0,AY779558:1.0):1.0,AY779563:1.0):1.0,AY779560:1.0):1.0,AY779561:1.0):1.0,AY779559:1.0):1.0):0.0,(((((AY786790:1.0,AY786791:1.0,AY786794:1.0,AY786797:1.0):1.0,(AY786792:1.0,(AY786793:1.0,AY786795:1.0,AY786796:1.0,AY786798:1.0,AY786799:1.0):1.0):1.0):1.0,((AY786800:1.0,AY786801:1.0,AY786802:1.0,AY786803:1.0,AY786804:1.0,AY786805:1.0,AY786806:1.0,(AY786807:1.0,AY786809:1.0):1.0,AY786808:1.0):1.0,(AY786810:1.0,AY786811:1.0,AY786812:1.0,AY786813:1.0,AY786814:1.0,AY786815:1.0,AY786816:1.0,AY786817:1.0,AY786818:1.0,AY786819:1.0,((AY786820:1.0,AY786822:1.0):1.0,((AY786821:1.0,AY786827:1.0,AY786829:1.0):1.0,AY786823:1.0,AY786824:1.0,AY786826:1.0,AY786828:1.0):1.0,AY786825:1.0):1.0):1.0):1.0):0.0,(JF320615:3.0,JF320617:3.0,JF320620:3.0,JF320621:3.0,JF320623:3.0,JF320624:3.0,JF320625:3.0,JF320626:3.0,JF320627:3.0):3.0):0.0,((AY786830:1.0,AY786831:1.0,AY786832:1.0,AY786833:1.0,AY786834:1.0,(AY786835:1.0,AY786837:1.0):1.0,AY786836:1.0,AY786838:1.0,AY786839:1.0):1.0,((((AY786840:1.0,AY786841:1.0,(AY786842:1.0,AY786847:1.0):1.0,AY786843:1.0,(AY786844:1.0,AY786848:1.0):1.0,AY786845:1.0,AY786849:1.0):1.0,AY786852:1.0):1.0,AY786854:1.0):1.0,AY786846:1.0,AY786851:1.0,((AY786853:1.0,AY786856:1.0):1.0,AY786869:1.0):1.0,AY786860:1.0,((AY786861:1.0,AY786864:1.0,AY786868:1.0):1.0,AY786867:1.0):1.0,(AY786863:1.0,AY786866:1.0):1.0,(AY786865:1.0,AY786858:1.0,AY786850:1.0):1.0,((AY786859:1.0,AY786857:1.0):1.0,AY786855:1.0,AY786862:1.0):1.0):1.0):0.0):0.0,(EF363125:2.0,EF363126:2.0):2.0,((EU807832:2.0,((EU807833:2.0,EU807834:2.0,EU807835:2.0,EU807836:2.0,EU807838:2.0):2.0,EU807837:2.0):2.0):0.0,(DQ487190:3.0,DQ487191:3.0):3.0):0.0,((FJ495937:2.0,FJ495939:2.0,FJ495940:2.0,FJ495941:2.0,FJ495942:2.0,FJ495943:2.0,((FJ495957:2.0,FJ495999:2.0):2.0,FJ495977:2.0,FJ495992:2.0,FJ495995:2.0,FJ495996:2.0,FJ495997:2.0,FJ495998:2.0):2.0,(FJ495958:2.0,((FJ495961:2.0,FJ495963:2.0):2.0,FJ495962:2.0):2.0,FJ495976:2.0,FJ495991:2.0,FJ495993:2.0):2.0,FJ495973:2.0,FJ495974:2.0,FJ495975:2.0,FJ495978:2.0,FJ495979:2.0,FJ495980:2.0,FJ495981:2.0,FJ495994:2.0):2.0,(FJ496000:2.0,FJ496001:2.0,FJ496002:2.0,FJ496003:2.0,FJ496004:2.0,FJ496005:2.0,(FJ496006:2.0,FJ496058:2.0):2.0,FJ496007:2.0,FJ496024:2.0,((FJ496025:2.0,(FJ496033:2.0,(FJ496034:2.0,FJ496036:2.0):2.0,FJ496035:2.0,FJ496037:2.0,FJ496038:2.0,FJ496040:2.0,FJ496041:2.0,(FJ919955:2.0,FJ919956:2.0,(((FJ919957:2.0,FJ919962:2.0):2.0,FJ919961:2.0):2.0,FJ919960:2.0):2.0,FJ919958:2.0,FJ919959:2.0):2.0):2.0):2.0,FJ496027:2.0,FJ496039:2.0):2.0,FJ496026:2.0,FJ496059:2.0,FJ496068:2.0,FJ496069:2.0,FJ496070:2.0,FJ496071:2.0,FJ496123:2.0,FJ496128:2.0,FJ496130:2.0,FJ496131:2.0,FJ496132:2.0,FJ496133:2.0,FJ496134:2.0,FJ496135:2.0,FJ496136:2.0):2.0):0.0,(((JF320363:2.0,JF320364:2.0,JF320366:2.0,JF320519:2.0):2.0,JF320365:2.0,JF320369:2.0,JF320373:2.0,JF320374:2.0,JF320514:2.0,JF320515:2.0,JF320516:2.0,JF320518:2.0):2.0,(HM208363:3.0,(JF320381:3.0,JF320384:3.0):3.0):0.0):0.0,(((JF320559:2.0,JF320561:2.0,JF320562:2.0,JF320563:

2.0):2.0,((((JF320460:3.0,JF320464:3.0):3.0,((JF320461:3.0,JF320468:3.0):3.0,JF320462:3.0,JF320463:3.0,JF320465:3.0,JF320466:3.0):3.0,JF320467:3.0):3.0,JF320469:3.0):3.0,JF320470:3.0):3.0):0.0,(JF320028:3.0,JF320029:3.0,JF320031:3.0,JF320032:3.0):3.0):0.0,(((((JF320044:3.0,JF320047:3.0,JF320049:3.0,JF320051:3.0,JF320062:3.0):3.0,JF320064:3.0):3.0,((JF320053:3.0,JF320058:3.0,JF320060:3.0):3.0,JF320055:3.0):3.0):3.0,(JF320145:3.0,JF320147:3.0,JF320152:3.0,JF320153:3.0,JF320154:3.0):3.0):0.0,((JF320048:3.0,JF320056:3.0,JF320065:3.0,JF320068:3.0,JF320071:3.0):3.0,(JF320179:3.0,JF320181:3.0,JF320182:3.0):3.0):0.0,((JF320059:3.0,(JF320307:3.0,JF320309:3.0,JF320311:3.0):3.0):0.0,((JF320197:3.0,JF320200:3.0,JF320202:3.0,JF320207:3.0):3.0,JF320205:3.0):3.0):0.0):0.0,((JF320183:2.0,(JF320187:2.0,JF320209:2.0,JF320212:2.0):2.0):2.0,(JF320186:3.0,JF320193:3.0,JF320201:3.0,JF320215:3.0):3.0):0.0,(((((JF320409:3.0,JF320569:3.0,JF320582:3.0):3.0,JF320591:3.0):3.0,(JF320571:3.0,JF320577:3.0):3.0,JF320572:3.0):3.0,JF320575:3.0):3.0,(JF320573:3.0,JF320574:3.0,JF320576:3.0):3.0):3.0):0.0);

| **T3** |
| --- |

(AY331282:1.0,AY331283:1.0,(((AY331284:1.0,AY332236:1.0):1.0,((AY331287:1.0,((HM586191:3.0,HM586193:3.0,HM586194:3.0):3.0,HM586196:3.0):3.0):0.0,(JF320185:3.0,JF320188:3.0,JF320190:3.0,JF320192:3.0,JF320194:3.0):3.0):0.0):0.0,(AY331285:1.0,AY331286:1.0):1.0,((AY331289:1.0,AY331290:1.0):1.0,((AY786880:1.0,(AY786882:1.0,AY786885:1.0):1.0,AY786888:1.0):1.0,(((AY786881:1.0,(AY786886:1.0,AY786887:1.0):1.0):1.0,AY786884:1.0):1.0,(AY786890:1.0,AY786891:1.0,(AY786892:1.0,AY786893:1.0,AY786895:1.0,AY786896:1.0,AY786899:1.0):1.0,AY786894:1.0,AY786897:1.0,AY786898:1.0):1.0):1.0,(AY786883:1.0,AY786889:1.0):1.0,((AY786900:1.0,AY786901:1.0):1.0,(AY786903:1.0,AY786904:1.0):1.0):1.0,AY786902:1.0):1.0):0.0,AY331293:1.0,(AY331296:1.0,AY331297:1.0):1.0,(AY423381:1.0,AY423382:1.0,(AY423384:1.0,(AY423385:1.0,AY423386:1.0):1.0):1.0):1.0,((((AY779550:1.0,AY779551:1.0):1.0,AY779564:1.0):1.0,AY779552:1.0):1.0,((((((AY779557:1.0,AY779562:1.0):1.0,AY779558:1.0):1.0,AY779563:1.0):1.0,AY779560:1.0):1.0,AY779561:1.0):1.0,AY779559:1.0):1.0):0.0,(((((AY786790:1.0,AY786791:1.0,AY786794:1.0,AY786797:1.0):1.0,(AY786792:1.0,(AY786793:1.0,AY786795:1.0,AY786796:1.0,AY786798:1.0,AY786799:1.0):1.0):1.0):1.0,((AY786800:1.0,AY786801:1.0,AY786802:1.0,AY786803:1.0,AY786804:1.0,AY786805:1.0,AY786806:1.0,(AY786807:1.0,AY786809:1.0):1.0,AY786808:1.0):1.0,(AY786810:1.0,AY786811:1.0,AY786812:1.0,AY786813:1.0,AY786814:1.0,AY786815:1.0,AY786816:1.0,AY786817:1.0,AY786818:1.0,AY786819:1.0,((AY786820:1.0,AY786822:1.0):1.0,((AY786821:1.0,AY786827:1.0,AY786829:1.0):1.0,AY786823:1.0,AY786824:1.0,AY786826:1.0,AY786828:1.0):1.0,AY786825:1.0):1.0):1.0):1.0):0.0,(JF320615:3.0,JF320617:3.0,JF320620:3.0,JF320621:3.0,JF320623:3.0,JF320624:3.0,JF320625:3.0,JF320626:3.0,JF320627:3.0):3.0):0.0,((AY786865:1.0,AY786858:1.0,AY786850:1.0):1.0,(((AY786840:1.0,AY786841:1.0,(AY786842:1.0,AY786847:1.0):1.0,AY786843:1.0,(AY786844:1.0,AY786848:1.0):1.0,AY786845:1.0,AY786849:1.0):1.0,AY786852:1.0):1.0,AY786854:1.0):1.0,AY786846:1.0,AY786851:1.0,((AY786853:1.0,AY786856:1.0):1.0,AY786869:1.0):1.0,AY786860:1.0,(AY786861:1.0,AY786864:1.0,AY786868:1.0):1.0,(AY786863:1.0,AY786866:1.0):1.0,AY786867:1.0,((AY786859:1.0,AY786857:1.0):1.0,AY786855:1.0,AY786862:1.0):1.0):1.0):0.0,((EF363125:2.0,EF363126:2.0):2.0,(FJ495937:2.0,FJ495939:2.0,FJ495940:2.0,FJ495941:2.0,FJ495942:2.0,FJ495943:2.0,((FJ495957:2.0,FJ495999:2.0):2.0,FJ495977:2.0,FJ495992:2.0,FJ495995:2.0,FJ495996:2.0,FJ495997:2.0,FJ495998:2.0):2.0,(FJ495958:2.0,((FJ495961:2.0,FJ495963:2.0):2.0,FJ495962:2.0):2.0,FJ495976:2.0,FJ495991:2.0,FJ495993:2.0):2.0,FJ495973:2.0,FJ495974:2.0,FJ495975:2.0,FJ495978:2.0,FJ495979:2.0,FJ495980:2.0,FJ495981:2.0,FJ495994:2.0):2.0):0.0,((EU807832:2.0,((EU807833:2.0,EU807834:2.0,EU807835:2.0,EU807836:2.0,EU807838:2.0):2.0,EU807837:2.0):2.0):0.0,(DQ487190:3.0,DQ487191:3.0):3.0):0.0,(FJ496000:2.0,FJ496001:2.0,FJ496002:2.0,FJ496003:2.0,FJ496004:2.0,FJ496005:2.0,(FJ496006:2.0,FJ496058:2.0):2.0,FJ496007:2.0,FJ496024:2.0,((FJ496025:2.0,((FJ496033:2.0,(FJ496034:2.0,FJ496036:2.0):2.0,FJ496038:2.0,FJ496041:2.0,(FJ919955:2.0,FJ919956:2.0,(((FJ919957:2.0,FJ919962:2.0):2.0,FJ919961:2.0):2.0,FJ919960:2.0):2.0,FJ919958:2.0,FJ919959:2.0):2.0):2.0,FJ496035:2.0,FJ496037:2.0,FJ496040:2.0):2.0):2.0,FJ496027:2.0,FJ496039:2.0):2.0,FJ496026:2.0,FJ496059:2.0,FJ496068:2.0,FJ496069:2.0,FJ496070:2.0,FJ496071:2.0,FJ496123:2.0,FJ496128:2.0,FJ496130:2.0,FJ496131:2.0,FJ496132:2.0,FJ496133:2.0,FJ496134:2.0,FJ496135:2.0,FJ496136:2.0):2.0,(((JF320363:2.0,JF320364:2.0,JF320366:2.0,JF320519:2.0):2.0,JF320365:2.0,JF320369:2.0,JF320373:2.0,JF320374:2.0,JF320514:2.0,JF320515:2.0,JF320516:2.0,JF320518:2.0):2.0,(HM208363:3.0,(JF320381:3.0,JF320384:3.0):3.0):0.0):0.0,(((JF320559:2.0,JF320561:2.0,JF320562:2.0,JF320563:2.0):2.0,(JF320186:3.0,JF320193:3.0,JF320201:3.0,JF320215:3.0):3.0):0.0,(JF320028:3.0,JF320029:3.0,JF320031:3.0,JF320032:3.0):3.0):0.0,(((((JF320044:3.0,JF320047:3.0,JF320049:3.0,JF320051:3.0,JF320062:3.0):3.0,JF320064:3.0):3.0,((JF320053:3.0,JF320058:3.0,JF320060:3.0):3.0,JF320055:3.0):3.0):3.0,(JF320145:3.0,JF320147:3.0,JF320152:3.0,JF320153:3.0,JF320154:3.0):3.0):0.0,((JF320048:3.0,JF320056:3.0,JF320065:3.0,JF320068:3.0,JF320071:3.0):3.0,(JF320179:3.0,JF320181:3.0,JF320182:3.0):3.0):0.0,((JF320059:3.0,(JF320307:3.0,JF320309:3.0,JF320311:3.0):3.0):0.0,((JF320197:3.0,JF320200:3.0,JF320202:3.0,JF320207:3.0):3.0,JF320205:3.0):3.0):0.0):0.0,(JF320183:2.0,(JF320187:2.0,JF320209:2.0,JF320212:2.0):2.0):2.0,((((JF320409:3.0,JF320569:3.0,JF320582:3.0):3.0,JF320591:3.0):3.0,(JF320571:3.0,JF320577:3.0):3.0,JF320572:3.0,JF320575:3.0):3.0,(JF320573:3.0,JF320574:3.0,JF320576:3.0):3.0):3.0,((((JF320460:3.0,JF320464:3.0):3.0,((JF320461:3.0,JF320468:3.0):3.0,JF320462:3.0,JF320463:3.0,JF320465:3.0,JF320466:3.0):3.0,JF320467:3.0):3.0,JF320469:3.0):3.0,JF320470:3.0):3.0):0.0);
