# Supplementary material for: Detecting consistent patterns of directional adaptation using differential selection codon models
Source: BMC Evol Biol. 2017 Jun 23;17:147. doi: 10.1186/s12862-017-0979-y (PMC5481935; doi:10.1186/s12862-017-0979-y)
Supplement: Supplementary file 3 — Figure S1. Posterior mean differential selection factors for all amino acids at all sites for two independent runs, for within-patients (a), B57+ patients (b) and B35+ patients (c). The correlation coefficient R2 is provided for each plot. (DOCX 112 kb) [file 12862_2017_979_MOESM3_ESM.docx]

**a**

| 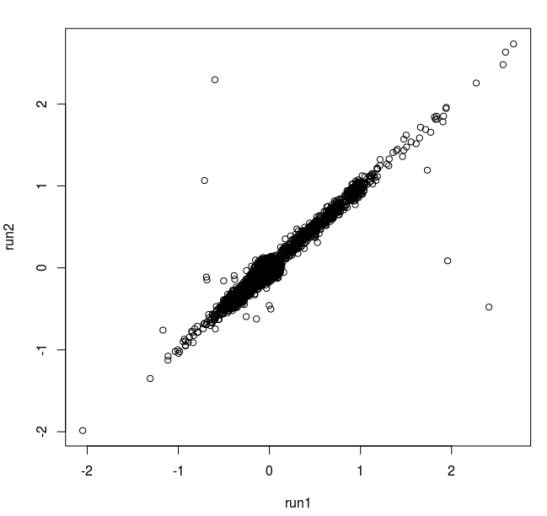  **b**  R^2^= 93% |
| --- |
| 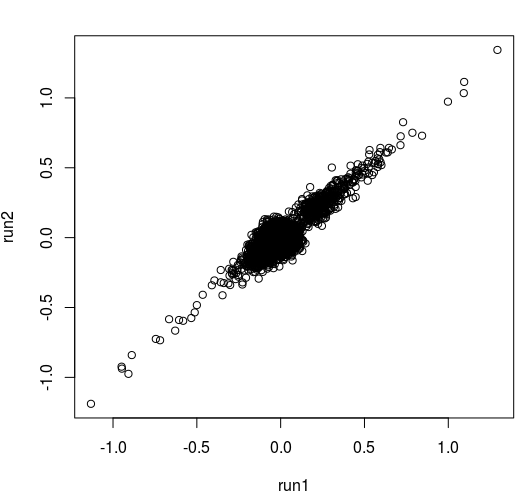  **c**  R^2^= 83% |
| 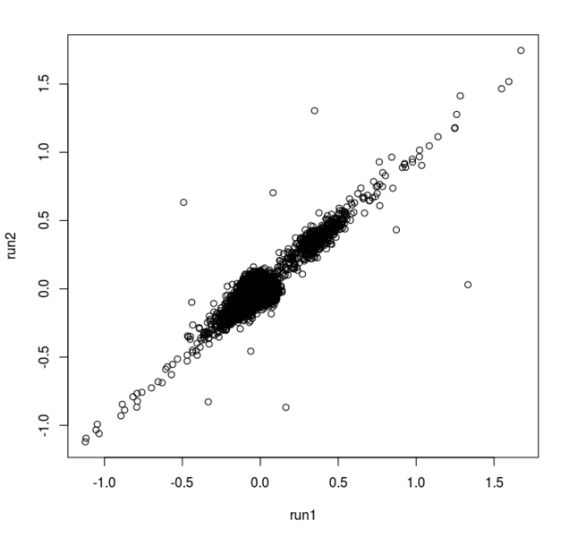  R^2^= 87% |

Figure S 1. Posterior mean differential selection factors for all amino acids at all sites for two independent runs, for within-patients (a), B57+ patients (b) and B35+ patients (c). The correlation coefficient R^2^ is provided for each plot.
